# Distinct transcriptional profiles of *Leptospira borgpetersenii* serovar Hardjo strains JB197 and HB203 cultured at different temperatures

**DOI:** 10.1371/journal.pntd.0009320

**Published:** 2021-04-07

**Authors:** Ellie J. Putz, Sathesh K. Sivasankaran, Luis G. V. Fernandes, Brian Brunelle, John D. Lippolis, David P. Alt, Darrell O. Bayles, Richard L. Hornsby, Jarlath E. Nally

**Affiliations:** 1 Infectious Bacterial Disease Research Unit, USDA Agriculture Research Service, National Animal Disease Center, Ames, Iowa, United States of America; 2 Food Safety and Enteric Pathogens Research Unit, USDA Agriculture Research Service, National Animal Disease Center, Ames, Iowa, United States of America; 3 Genome Informatics Facility Iowa State University, Ames, Iowa, United States of America; 4 Laboratório de Desenvolvimento de Vacinas, Instituto Butantan, São Paulo, Brazil; 5 Arbor Biosciences, Ann Arbor, Michigan, United States of America; 6 Ruminant Disease and Immunology Research Unit USDA Agriculture Research Service, National Animal Disease Center, Ames, Iowa, United States of America; Universidade Federal de Pelotas, BRAZIL

## Abstract

**Background:**

Leptospirosis is a zoonotic, bacterial disease, posing significant health risks to humans, livestock, and companion animals around the world. Symptoms range from asymptomatic to multi-organ failure in severe cases. Complex species-specific interactions exist between animal hosts and the infecting species, serovar, and strain of pathogen. *Leptospira borgpetersenii* serovar Hardjo strains HB203 and JB197 have a high level of genetic homology but cause different clinical presentation in the hamster model of infection; HB203 colonizes the kidney and presents with chronic shedding while JB197 causes severe organ failure and mortality. This study examines the transcriptome of *L*. *borgpetersenii* and characterizes differential gene expression profiles of strains HB203 and JB197 cultured at temperatures during routine laboratory conditions (29°C) and encountered during host infection (37°C).

**Methodology/Principal findings:**

*L*. *borgpetersenii* serovar Hardjo strains JB197 and HB203 were isolated from the kidneys of experimentally infected hamsters and maintained at 29°C and 37°C. RNAseq revealed distinct gene expression profiles; 440 genes were differentially expressed (DE) between JB197 and HB203 at 29°C, and 179 genes were DE between strains at 37°C. Comparison of JB197 cultured at 29°C and 37°C identified 135 DE genes while 41 genes were DE in HB203 with those same culture conditions. The consistent differential expression of *ligB*, which encodes the outer membrane virulence factor LigB, was validated by immunoblotting and 2D-DIGE. Differential expression of lipopolysaccharide was also observed between JB197 and HB203.

**Conclusions/Significance:**

Investigation of the *L*. *borgpetersenii* JB197 and HB203 transcriptome provides unique insight into the mechanistic differences between acute and chronic disease. Characterizing the nuances of strain to strain differences and investigating the environmental sensitivity of *Leptospira* to temperature is critical to the development and progress of leptospirosis prevention and treatment technologies, and is an important consideration when serovars are selected and propagated for use as bacterin vaccines as well as for the identification of novel therapeutic targets.

## Author summary

Leptospirosis is a global zoonotic, neglected tropical disease. Interestingly, a high level of species specificity (both bacteria and host) plays a major role in the severity of disease presentation which can vary from asymptomatic to multi-organ failure. Pathogenic *Leptospira* colonize the kidneys of infected individuals and are shed in urine into the environment where they can survive until they are contracted by another host. This study looks at two strains of *L*. *borgpetersenii*, HB203 and JB197 which are genetically very similar, and identical by serotyping as serovar Hardjo, yet HB203 causes a chronic infection in the hamster while JB197 causes organ failure and mortality. To better characterize bacterial factors causing different disease outcomes, we examined the gene expression profile of these strains in the context of temperatures that would reflect natural *Leptospira* life cycles (environmentally similar 29°C and 37°C which is more indicative of host environment). We found vast differences in gene expression both between the strains and within strains between temperatures. Characterization of the transcriptome of *L*. *borgpetersenii* serovar Hardjo strains JB197 and HB203 provides insights into factors that can determine acute versus chronic disease in the hamster model of infection. Additionally, these studies highlight strain to strain variability within the same species, and serovar, at different growth temperatures, which needs to be considered when serovars are selected and propagated for use as bacterin vaccines used to immunize domestic animal species.

## Introduction

Global human leptospirosis incidence approaches an estimated 1.03 million annual cases, of which, almost 60,000 are estimated to be fatal [1]. *Leptospira* are shed in the urine of infected individuals, most commonly by reservoir hosts, and can be transmitted directly from animal to human or indirectly picked up from the environment. Disease presentation can vary from asymptomatic in reservoir hosts, to flu-like symptoms, and multi-organ failure in severe cases in incidental hosts [2]. *L*. *interrogans* is the leading cause of human infection but leptospirosis can affect most vertebrates including all major domestic livestock. In cattle, acute leptospirosis infections present with reproductive symptoms including abortion, embryonic loss, preterm calving, or low health calves [3, 4], which can result in devastating animal health and economic damages. A recent study found that 7.2% of beef cattle from an abattoir in the central United States were actively shedding *L*. *borgpetersenii* [5].

Including pathogenic and saprophytic species, there are 64 species of *Leptospira* identified and hundreds of serovars [6]. The presentation of disease severity and reservoir host status is highly specific between host species and the serovar/species of the infecting *Leptospira*. For instance, while rats have been identified as reservoir hosts of leptospirosis since the early 1900s [7], most prominently of serovars Icterohaemorrhagiae and Copenhageni, cattle are recognized as the true reservoir hosts of serovar Hardjo [4, 8]. A specific species of *Leptospira* may represent numerous serovars, and a single serovar can include numerous species, for example, serovar Hardjo can be represented by both species *L*. *borgpetersenii* (type Hardjo bovis) and *L*. *interrogans* (subtype Hardjo prajitno). Bacterin vaccines for leptospirosis lack cross protection between serogroups and have only weak cross protection across serovars [9, 10]. Thus, most vaccines are composed of numerous serovars, using those most common in the designated region and target species. In cattle, vaccination for serovar Hardjo is highly sensitive to *Leptospira* species, where vaccination by *L*. *borgpetersenii* and *L*. *interrogans* may not offer cross species protection [11–14]. In fact, for serovar Hardjo, species specific interactions do not stop at the serovar or species level. JB197 and HB203 are two different strains of species *L*. *borgpetersenii* serovar Hardjo both isolated from cattle at slaughter; HB203 was isolated from 10 year old dairy cow in Kansas, USA and JB197 was isolated from a bull in Nebraska, USA [15]. Both strains are highly similar by sequence homology and indistinguishable by serovar and MAT (microscopic agglutination test) [16]. Yet HB203 inoculation in the hamster model of leptospirosis results in colonization of the kidney and the establishment of a chronic asymptomatic infection. In contrast, JB197 challenge will result in severe acute disease, with *Leptospira* identifiable in the blood and tissue and severe damage inflicted across numerous organs, resulting in death of the hamster [16]. This evidence strongly suggests that host interactions and environmental response at the strain level are critical to the biological control or failure to control *Leptospira* infections.

In the context of vaccine development, much emphasis has been placed on identifying outer membrane proteins or virulence factors that could potentially confer cross protection between species and serovars. In Gram-negative bacteria, lipopolysaccharide (LPS) is one of the best characterized pathogen-associated molecule patterns (PAMPs) and is an obvious candidate target for agglutinating antibody production. *Leptospira* LPS is considerably less endotoxic than other Gram-negative LPS such as *Escherichia coli*, however, leptospiral LPS still elicits a protective antibody response against lethal challenge [17]. The variable O-antigen region of leptospiral LPS is synthesized by genes in the *rfb* locus, which in *L*. *borgpetersenii*, contains 31 open reading frames [18, 19]. Among other virulence candidates are the leptospiral immunoglobulin-like (Lig) proteins which have been established to have protective properties as immunogens. Expression of the Lig proteins are known to be influenced by osmolarity of their environment (increased at physiological osmolarity) and culture attenuation (decreased with serial culture passage) [20, 21]. While some species such as *L*. *interrogans* carry the genes encoding both LigA and LigB, *L*. *borgpetersenii* only contains LigB [22]. Notably, while concurrent knock down of LigA and LigB in *L*.*interrogans* resulted in attenuated virulence [23], in an alternative study where the *L*. *interrogans* LigB gene was disrupted, but the LigA was still functional, virulence was not diminished and colonization of the kidney was still accomplished [24]. While this study established that in *L*. *interrogans*, LigB was not essential for virulence it is possible that LigA has redundant functions, making *L*. *borgpetersenii* an increasingly interesting model for the study of LigB.

Temperature is a major factor leptospires must navigate within the environments they encounter over their lifecycle. Leptospirosis is most common in tropical environments where frequent rainfall, warm temperatures, and soil pH create optimum environments for the survival and environmental persistence of *Leptospira* [25]. This can be illustrated in the context of the natural lifecycle of *Leptospira*, which may be shed in the urine of an infected host (leaving an host kidney similar to 37°C) to a cooler, moist environment (soil, grass, forage, water source, etc. similar to 29°C) and then once more be picked up by a new host where it will ultimately strive to persist again in the kidney or other tissue at basal host temperature. In the laboratory, leptospires are almost exclusively cultured at 29°C, which potentially limits what behavior researchers are able to observe in conditions that do not best mimic a true *in vivo* environment. This is currently an area of progress in the leptospirosis research field as our group recently described methodology for successful primary culture of live organisms at 37°C utilizing a new HAN media formulation [26]. For the first time, this has enabled the long term culture propagation of *L*. *borgpetersenii* directly isolated from host tissue at 29°C or 37°C [26].

While the *L*. *borgpetersenii* serovar Hardjo strains JB197 and HB203 produce such divergent severity of disease phenotypes, we sought to examine the transcriptomic behavior of these two strains in the context of an additional *in vitro* factor: temperature. In this study we establish distinct differential gene expression profiles of JB197 and HB203 each cultured at both 29°C and 37°C. We identified distinct profiles between strains and within strain between temperatures, emphasizing the importance of strain to strain variation and the acute sensitivity of *Leptospira* to its environment.

## Methods

### Ethics statement and bacteria

All animal experimentation was conducted in accordance with protocols as reviewed and approved by the Animal Care & Use Committee at the National Animal Disease Center, and as approved by USDA Institutional guidelines. Two groups of three golden Syrian hamsters (*Mesocricetus auratus*) were inoculated with *L*. *borgpetersenii* serovar Hardjo strain HB203 or JB197 as previously described [16]. In the case of hamsters infected with JB197, kidney tissue was harvested when hamsters presented with clinically severe symptoms (blood on nose, pads, or urogenital tract, weight loss, dehydration, general poor condition, etc.) at approximately five days post-infection. In the case of hamsters infected with HB203, kidney tissue was harvested at three weeks post-infection. In both cases, kidney tissue was cultured to recover strains JB197 and HB203 in HAN medium incubated at 29 and 37°C, and as previously described [26]. Recovered isolates from a single kidney from each strain were sub-cultured into HAN liquid media at indicated temperatures and harvested by centrifugation (10,000 x *g*, 4°C, 30 min) at mid-late log phase of growth (~2 x 10^8^ leptospires/mL). Four biological replicates were utilized for RNA isolation and additional bacterial pellets were frozen down for additional analysis.

### RNA isolation

#### RNA samples submitted for RNAseq

Four replicates were treated with RNA Protect (Cat. No. 76506 Qiagen, MD, USA) following manufacturer’s directions. Bacterial pellets were resuspended in Lysozyme (Sigma Aldrich, Cat. No. L6876) TE solution and incubated at room temperature with continuous shaking. RNA was isolated following the RNeasy Mini Kit (Cat. No. 74104, Qiagen, MD, USA) and manufacturer’s instructions. RNA samples were treated with 80 units of Recombinant Ribonuclease Inhibitor RNase Out (5000U ThermoFisher, MA, USA). For DNA clean-up, samples were additionally treated with the Invitrogen Turbo DNA free kit (Cat. No. AM1907, ThermoFisher, MA, USA) to manufacturer’s specifications. For ribosomal depletion, samples were treated with the Ribo-Zero rRNA removal kit (Cat. No. MRZB12424, Illumina, Inc., San Diego, CA, USA) according to manufacturer’s guidelines.

#### RNA samples used for RT-qPCR

Frozen original bacterial pellets containing 2.5–5 x 10^8^ leptospires were later thawed and utilized for RT-qPCR validation using the Trizol method as previously described [27]. Briefly, 1 mL Trizol (Invitrogen, CA, USA) was added to frozen pellets and pipetted slowly until thawed. Samples were vortexed and incubated for 10 minutes at room temperature at which time, 260 uL chloroform was added, samples were vigorously shaken, and incubated for another 10 minutes at room temperature. Samples were centrifuged at 12,000 x *g* for 10 minutes at 4°C to achieve phase separation; the aqueous phase was collected and 660 uL isopropanol was added to facilitate nucleic acid precipitation. After 10 minutes incubation at room temperature samples were centrifuged at 12,000 x *g* for 10 minutes at 4°C whereafter resulting pellets were washed with 75% ethanol. Washed pellets were dried for 30 minutes using a Speedvac. Dry pellets were resuspended in 50 uL RNAse free water and incubated at 55°C for 10 minutes. For residual DNA removal, samples were additionally treated with the Turbo DNA free kit (Ambion, Cat. No. AM1907, TX, USA) to manufacturer’s specifications. RNA was examined for quality by Aligent Bioanalzyer and quantified using the Qubit RNA system (ThermoFisher, MA, USA) according to manufacturer’s directions.

### Sequencing

For sequencing, RNA was submitted to Iowa State University DNA Facility for library preparation using the TruSeq stranded total RNA kit (Cat. No. RS-122-2203, Illumina, Inc., San Diego, CA, USA) and sequenced using 150 Cycle single-read on an Illumina HiSeq 3000.

### Analysis & genome alignment

RNAseq reads were aligned to the JB197 reference genome (RefSeq ID: chromosome 1: NC_008510.1, chromosome 2: NC_008511.1) and/or the HB203 reference genome (RefSeq ID: chromosome 1: NZ_CP021412.1, chromosome 2: NZ_CP021413.1) using Segemehl [28] with default mapping parameters. For differential gene expression analysis between strains, reads from HB203 were mapped to the JB197. Uniquely aligned reads (reads aligned to only one location in genome) were only processed for the downstream analysis and reads were formatted graphically to visualize in the Integrated Genome Browser (IGB [29]). Read counts (number of reads that aligned to a specific gene) for each gene were quantified using in-house Perl scripts. The genomic sequences of JB197 and HB203 were compared using rapid dotter software GEPARD (http://cube.univie.ac.at/gepard) with “word-length” parameter 750 for chromosome 1 and 100 for chromosome 2.

### Statistics

Differential expression (DE) analysis was completed in R (v3.6.1; https://www.r-project.org/) using the DESeq2 package (v1.29.0 [30]). Briefly, raw read counts were normalized and reproducibility of the biological replicates was examined using Spearman rank correlation. Principle Component Analysis (PCA) was performed to determine outliers in the RNAseq dataset. Genes with normalized read counts less than 10 in less than three of the four replicates within a condition were dropped from DE analysis. To identify DE genes, four independent analyses (JB197 37°C vs. 29°C, HB203 37°C vs. 29°C, 29°C JB197 vs. HB203, and 37°C JB197 vs. HB203) were conducted using linear regression models within DESeq2. For a gene to be considered significantly differentially expressed, it was required to have an adjusted p-value < 0.05 and have a fold change (FC) greater than or equal to three. For JB197 versus HB203 comparison, positive fold change values indicate higher expression in JB197; between temperatures, 37°C vs. 29°C, positive values indicate upregulation at 37°C. PCA and heatmaps were created in R (version 3.6.1) [31]. Venn diagrams were constructed with R and Venny [32].

### RT-qPCR

Select genes were chosen for additional analysis and RNAseq validation by RT-qPCR. RNA was isolated and quantified as described above, and cDNA conversion was completed using the iScript Reverse Transcription Supermix (BioRad) and manufacturer’s instructions. Of the common bacterial RT-qPCR control genes that had been previously validated [33], most were differentially expressed in at least one of our primary contrasts of interest (29°C (JB197 vs. HB203), 37°C (JB197 vs. HB203), JB197 (37°C vs. 29°C), HB203 (37°C vs. 29°C)). As a result, two separate control genes were utilized. One control gene was *secA* that was not differentially expressed and was suitable as a control for 29°C (JB197 vs. HB203) and JB197 (29°C vs. 37°C) contrasts. The second control gene was *rho*, which was not differentially expressed and was suitable as a control for 37°C (JB197 vs. HB203) and HB203 (37°C vs. 29°C). RNA concentrations were normalized to the condition with the lowest amount of RNA present prior to cDNA conversion. RT-qPCR was performed in 20-μL reactions (1-μL cDNA, 400nM of each forward (F) and reverse (R) primers, and 10-μL of SYBR green PCR mix (BioRad)) under the following conditions; 50°C for 2 minutes, 95°C for 10 minutes, followed by 40 cycles of 95°C for 15 seconds and 60°C for 30 seconds. Primers used were as follows:

*ligB*: F’TGACGAGAATCGGGGATTAG, R’ACTGCCGTCCGAATAAACAC,
LipL45: F’ CAACAAGGCCTCCAAAGAAG, R’AATCGCAATTCGAGGAGCTA,
LBJ_RS02895: F’GCCGCTTTGAGCATTCTATC, R’AGTCCCACTACCCTGCATTG,
LBJ_RS11060: F’ CGCAGAACGAAAAGGAAGAC, R’AGGCTCGGAGAAGTCACAAA,
*secA*: F’ GGAGGAATCGCTCTTCACAG, R’ CGTCCCTCTTTGCGAGATAG,
*rho*: F’ TTTAAGAACCGGGGACACAG, R’ TTCCAATCGGACACATGAGA.

Prior to RT-qPCR, primers were tested by PCR and had primer efficiencies calculated. For all RT-qPCR runs matched reverse transcriptase negative (RT-) and no template controls were performed for quality control. The 2^−ΔΔCT^ method was used for data analysis, and error bars indicated in RT-qPCR figures represent 95% confidence intervals.

### 1 and 2-D gel electrophoresis and immunoblotting

Leptospires were harvested by centrifugation (10,000 x *g*, 4°C, 30 min), washed twice with PBS, and processed for one-dimensional (1-D) SDSPAGE on 12% acrylamide gels (BioRad, CA, USA) as per manufacturer’s guidelines. Proteins were visualized by staining with Sypro Ruby (Invitrogen,CA, USA) and lipopolysaccharide was visualized by staining with Pro-Q Emerald 300 (Invitrogen, CA, USA) as per manufacturer’s guidelines. For immunoblotting, samples were transferred by semi-dry transfer (Amersham TE77 PWR) to Immobilon-P transfer membrane (Millipore, 220 Bedford, MA, USA) and blocked overnight at 4°C with StartingBlock (PBS) blocking buffer (Thermo Scientific, MA, USA). Membranes were individually incubated with indicated antisera diluted in blocking buffer (anti-LigA/B at 1:5,000 or anti-LipL45 at 1:2,000) followed by incubation with horseradish-peroxidase anti-rabbit immunoglobulin G conjugate diluted 1:4,000 in blocking buffer (Sigma, MO, USA). Bound conjugates were detected using Clarity Western ECL substrate (BioRad, CA) and images acquired using a Bio-Rad ChemiDoc MP imaging system. Immunoblots were also performed on unwashed leptospires as used for isolation of RNA. Representative images of results are provided.

For 2-D DIGE, fractions enriched for outer membrane (OM) proteins were prepared from strain JB197 or HB203, cultured at both 29 and 37°C, in triplicate, using Triton X-114 as previously described [34]. OM enriched fractions were resuspended in solubilization buffer (7M Urea, 2M Thiourea, 1% ASB-14) and quantified using the RC DC protein assay (BioRad, CA, USA). Protein samples (20 μg) were labeled with Cy3 or Cy5 (GE Healthcare, IL, USA) for comparison as indicated in **Table 1**; for each gel, 20 μg of internal standard, comprising equal μg amounts of all 12 replicates, were labelled with Cy2. 2-D gel electrophoresis of labeled samples was performed using 24cm IPG strips, pH 3–10, as previously described [35]. Gels were scanned using the Typhoon fluorescence gel scanner (Amersham) using different band-pass filters (520 nm for Cy2, 580 nm for Cy3 and 670 nm for Cy5) to image each of the three CyDyes (GE Healthcare, IL, USA). Differential protein expression was analyzed using SameSpots (TotalLab, UK) following the software manual. The differentially expressed protein spot of interest (corresponding to predicted mass of LigB) was excised from a master gel comprising OM enriched fraction of HB203 at 29°C and processed for identification by mass spectrometry using the In-gel Tryptic digestion kit and C18 Spin columns (ThermoFisher Scientific, MA, USA) as per manufacturer’s instructions.

**Table 1:** Experimental design for DIGE experiment. Three replicates (A, B & C) of *L*. *borgpetersenii* serovar Hardjo strain HB203 and JB197, cultured at 29 or 37°C, were labeled with Cy3 or Cy5. An internal control comprising an equal amount of all 12 replicates was labeled with Cy2.

Gel #	Cy3	Cy5	Cy2
1	HB203, 29°C, A	JB197, 29°C, A	Internal control
2	JB197, 37°C, A	HB203, 37°C, A	Internal control
3	HB203, 37°C, B	JB197, 29°C, B	Internal control
4	JB197, 37°C, B	HB203, 29°C, B	Internal control
5	HB203, 29°C, C	JB197, 37°C, C	Internal control
6	JB197, 29°C, C	HB203, 37°C, C	Internal control

### LC-MSMS and database searching

Peptides from the gel spot were separated by HPLC chromatography using a Proxeon Easy-nLC (Thermo Fisher Scientific, West Palm Beach, FL, USA) connected to the mass spectrometer. The chromatography used a trapping column (Proxeon Easy-Column, 2 cm, ID 100 μm, 5um, 120A, C18) and an analytical column (Proxeon Easy-Column, 10 cm, ID 75 μm, 3um, 120A, C18). The gradient used a mobile phase A (95% H_2_O: 5% acetonitrile and 0.1% formic acid) and mobile phase B (5% H_2_O: 95% acetonitrile and 0.1% formic acid). The analytical column was connected to a PicoTip Emitter (New Objectives, Woburn, MA, USA; FS360-75-15-N-20) cut to size. The column and Emitter were attached to a LTQ OrbiTrap Velos Pro (Thermo Fisher Scientific, West Palm Beach, FL, USA) mass spectrometer using the Proxeon Nanospray Flex Ion Source. The capillary temperature was set at 275°C and spray voltage was 2.4 kV. The mass spectrometer used a data dependent method. In MS mode the instrument was set to scan 300–2000 m/z with a resolution of 60,000 FWHM. A minimal signal of 10,000 could trigger MSMS and 10 consecutive MSMS were possible. The activation type used was CID. The normalized collision energy was set to 35 and repeat mass exclusion was set to 120 seconds.

Tandem mass spectra were analyzed using Proteome Discoverer version 2.2. All MS/MS samples were analyzed using Sequest HT (Thermo Fisher Scientific, CA, USA) assuming digestion with trypsin. Proteome Discoverer search used a fasta database downloaded from UniProt. The fasta database was generated by searching Uniprot with the criteria “Hardjo”, then limiting the database using the UniRef 90% feature [36]. The Hardjo UniRef90 fasta file was generated in July 2020 and consisted of 8312 entries. The mass spectrometer.raw file was searched with a fragment ion mass tolerance of 0.6 Da, a parent ion tolerance of 10.0 PPM, and allowed 2 missed cleavages. Carbamidomethyl of cysteine was specified as fixed modifications. Deamidation of asparagine and glutamine, and oxidation of methionine were specified as variable modifications.

## Results

### Different protein profiles distinguish *L*. *borgpetersenii* serovar Hardjo strain JB197 compared to strain HB203

**Fig 1:**
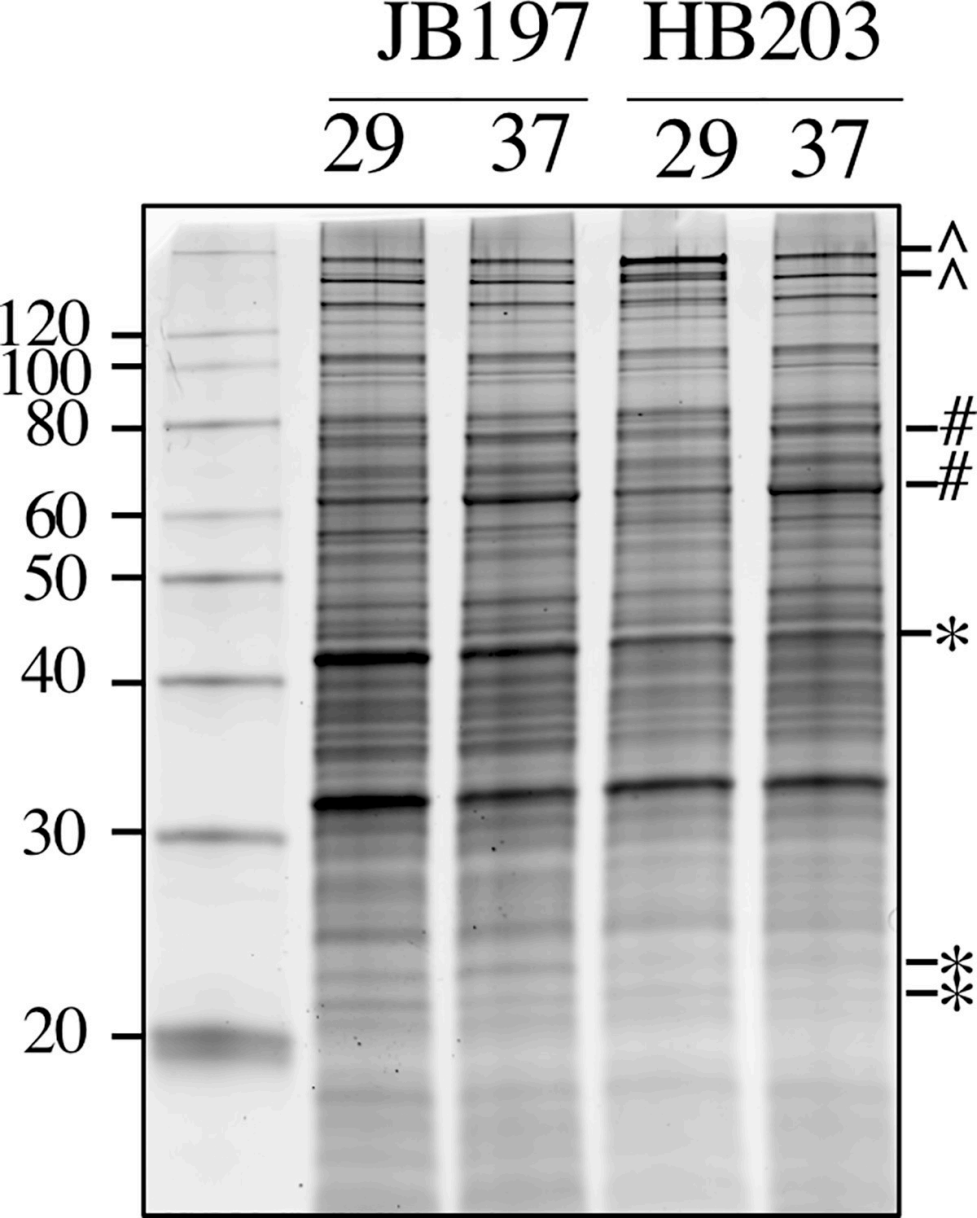
Total protein (5μg) produced by serovar Hardjo strain JB197 and strain HB203 at 29 or 37°C. *indicates proteins increased in JB197 compared to HB203. ^indicates proteins increased in HB203 compared to JB197. ^#^indicates proteins increased at 37 compared to 29°C. Molecular mass markers (kDa) are indicated.

Strains JB197 and HB203 are indistinguishable by serovar testing, but significant variation in disease presentation in the hamster model point to meaningful differences that may impact host recognition or immune escape by the pathogen. Strain JB197 and strain HB203 were cultured from the kidney of hamsters with severe acute, or persistent chronic leptospirosis respectively and as previously described [16], and culture maintained at 29 or 37°C. Serovar Hardjo is highly fastidious but the development of a new media, designated HAN media, has enabled serovar Hardjo to be cultured directly from a mammalian host at 29 or 37°C [26]. Comparative analysis of protein content suggests that different protein profiles are expressed by strain JB197 compared to HB203 at both 29 and 37°C, **Fig 1**.

### Comparative genome analysis of serovar Hardjo strain JB197 and strain HB203

The complete genome comprising both chromosomes of *L*. *borgpetersenii* serovar Hardjo strain JB197 and strain HB203 were compared by genome alignment, **S1 Fig**. Both chromosome 1 and chromosome 2 were highly similar between strains (NCBI BLAST percent identity = 99.85% for chromosome I, and 99.90% for chromosome II). As previously described for serovar Hardjo strain JB197 compared to serovar Hardjo strain L550, an inversion in the middle of chromosome I differentiates strain JB197 from HB203, **S1A Fig**.

### The transcriptome of serovar Hardjo strain JB197 and HB203

**Fig 2:**
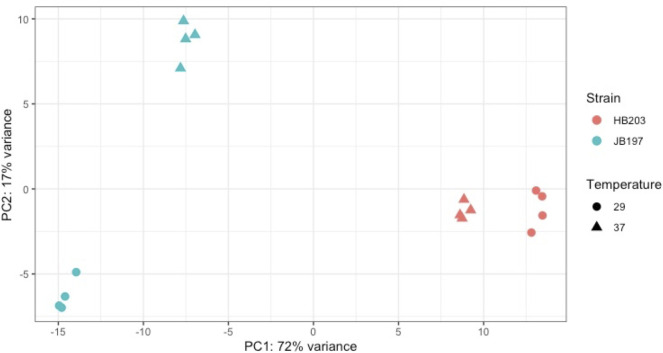
Principle Component Analysis (PCA) of strain and temperature.

Several factors contributed to our interest in the transcriptome of strains JB197 and HB203 at 29 and 37°C. First, serovar Hardjo strain JB197 causes an acute lethal disease in experimentally infected hamsters compared to serovar Hardjo strain HB203 which causes a persistent renal colonization. Second, the ability to culture serovar Hardjo directly from hamster tissue in HAN media at 29 or 37°C has only recently become possible [26], and thirdly, there was evidence of differential protein expression between and within strains under different growth temperatures at the total protein level (**Fig 1**). RNAseq was performed on four biological replicates of each strain and temperature condition. Mapping details including uniquely and total mapped reads, are presented in **S1 Table**. Principle Component Analysis (PCA) indicated that strain and temperature collectively explained 89% of total variation (PC1 = 72%, PC2 = 17%) (**Fig 2)**, and that our RNAseq biological replicates within each condition were robust and highly reproducible (**Fig 2**, Spearman correlations seen in **S2 Fig)**. While all datasets clustered closely to their respective strain and growth temperature conditions, it was clear that HB203 at 29°C and HB203 at 37°C clustered much more tightly to each other than JB197 at 29°C and JB197 at 37°C, suggesting greater variation between stains, and that HB203 exhibited less of a global difference due to temperature.

To explore the effects of strain and temperature on gene expression profiles, differential expression (DE) of four main contrasts of experimental groups were analyzed. The first contrast examined differences between the two strains cultured at the classic leptospiral isolation and cultivation temperature of 29°C: JB197 29°C vs. HB203 29°C. The second contrast examined strain differences cultured at the newly achieved 37°C temperature: JB197 37°C vs. HB203 37°C. Since such diverse clinical signs of disease are seen in the hamster model between the acute lethal infection caused by JB197 and the chronic presentation of HB203, the third and fourth contrasts captured the effect of temperature on each strain individually, JB197 37°C vs. JB197 29°C, and HB203 37°C vs. HB203 29°C, respectively.

**Fig 3:**
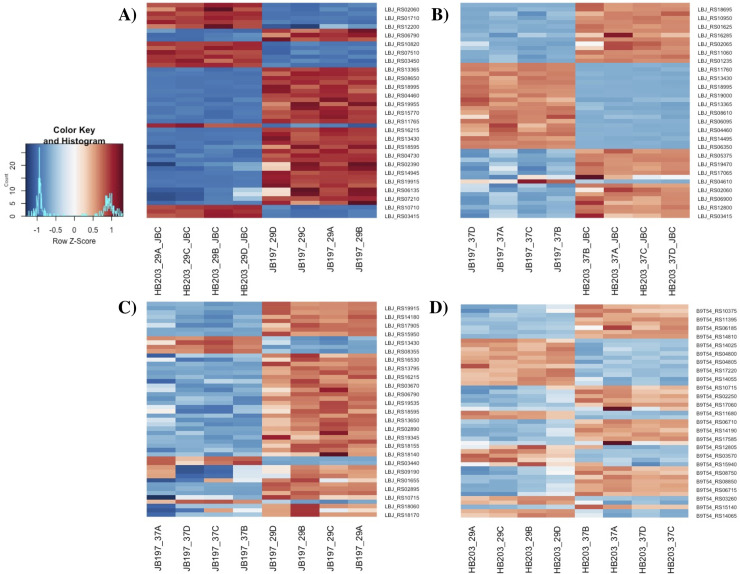
Heatmaps illustrating the top 25 genes with different expression (by Z-score) of major contrasts of interest. (A) JB197 vs. HB203 cultured at 29°C, (B) JB197 vs. HB203 cultured at 37°C, (C) JB197 cultured at 29°C vs. 37°C and (D) HB203 cultured at 29°C vs. 37°C. All four biological replicates are shown for each contrast.

To broadly visually illustrate gene DE in our contrasts of interest, heatmap profiles of the top 25 genes showing the most difference in overall expression (by Z-score) of each of these contrasts are presented, **Fig 3**. Differences in transcriptomic profiles are prominent in each of the major contrasts of interest. In agreement with the PCA plot, color intensity indicative of Z-score indicates stronger expression differences between JB197 and HB203 at 29°C, **Fig 3A**, and the least amount of variation from the mean gene transcript levels in HB203 between 29°C and 37°C, **Fig 3D**.

The DESeq2 package was used to identify genes that were significantly differentially expressed with a minimum fold change (FC) of three and adjusted p-value < 0.05 (adj. p-value) in each of the major contrasts of interest. For the 29°C contrast between strain JB197 vs. HB203, 440 DE genes were identified, the fifty most significant of which are reported in **Table 2**, and the complete list in **S2 Table**. Analysis of JB197 and HB203 cultured at 37°C identified 179 DE genes, the fifty most significant of which are reported in **Table 3** and the complete list in **S3 Table**. Within strain JB197, 135 DE genes were identified between 37°C and 29°C growth conditions, the fifty most significant of which are reported in **Table 4** and the full list can be viewed in **S4 Table**. Within HB203, 41 DE genes were identified between 37°C and 29°C growth conditions, all of which are reported in **Table 5**; the complete list of DE genes, including those not significantly DE expressed by a three-fold change minimum for this contrast, are reported in **S5 Table**.

**Table 2:** The fifty most significantly differentially expressed genes between JB197 and HB203 grown at 29°C. All genes are relative to the JB197 reference genome and annotated by the JB197 gene ID. * Positive fold change values denote higher transcription in JB197 and conversely, negative values indicate higher expression in HB203.

No.	Gene ID	Description	Adjusted p-value	Fold Change *
1	LBJ_RS01710	hypothetical protein	0	-10.4004071
2	LBJ_RS19915	hypothetical protein	0	71.87672896
3	LBJ_RS14945	hypothetical protein	0	189.7898994
4	LBJ_RS02895	hypothetical protein	0	220.5420141
5	LBJ_RS03430	hypothetical protein	2.4E-281	-11.1548737
6	LBJ_RS03415	Lipoprotein LigB	8.9E-278	-54.1029728
7	LBJ_RS15950	hypothetical protein	9.1E-277	99.91598353
8	LBJ_RS08135	heavy metal translocating P-type ATPase	1.1E-274	-11.767568
9	LBJ_RS03420	hypothetical protein	2.4E-271	-52.2580436
10	LBJ_RS11760	AMP-binding protein	5.4E-239	68.74086102
11	LBJ_RS12405	response regulator	2.8E-235	-8.98517579
12	LBJ_RS08130	hypothetical protein	1.1E-229	-11.5917582
13	LBJ_RS05570	EAL domain-containing response regulator	2.1E-227	-6.63290887
14	LBJ_RS10820	IS110 family transposase	4.4E-223	-20.9247722
15	LBJ_RS01700	SpoIIE family protein phosphatase	1.8E-201	-5.49377076
16	LBJ_RS03425	hypothetical protein	1.9E-201	-17.6651623
17	LBJ_RS05565	response regulator	2.2E-192	-9.00271506
18	LBJ_RS08110	cbb3-type cytochrome c oxidase subunit II	3.3E-189	-7.37618465
19	LBJ_RS08105	cytochrome-c oxidase%2C cbb3-type subunit I	3.3E-189	-6.81110652
20	LBJ_RS12400	hypothetical protein	6.2E-184	-9.4793474
21	LBJ_RS14935	penicillin acylase family protein	1.4E-182	6.460520742
22	LBJ_RS08145	sulfite exporter TauE/SafE family protein	5.7E-182	-11.0983029
23	LBJ_RS08740	RNA-binding protein	1.2E-175	-3.71297255
24	LBJ_RS14495	IS110 family transposase	2.6E-174	16.05882712
25	LBJ_RS10950	DUF1761 domain-containing protein	1.7E-168	-6.80842599
26	LBJ_RS10710	hypothetical protein	3.1E-166	-20.8405012
27	LBJ_RS11060	hypothetical protein	4E-165	-20.3398952
28	LBJ_RS12655	hypothetical protein	3.4E-161	-3.29637117
29	LBJ_RS08125	cytochrome c oxidase accessory protein CcoG	3.4E-158	-8.95401048
30	LBJ_RS01505	hypothetical protein	6E-157	-5.01089261
31	LBJ_RS08120	c-type cytochrome	1.7E-155	-5.6523847
32	LBJ_RS03005	methyl-accepting chemotaxis protein	3E-149	-5.11212153
33	LBJ_RS16975	hypothetical protein	1E-143	-6.17144957
34	LBJ_RS05970	methyl-accepting chemotaxis protein	3.1E-143	-5.96096974
35	LBJ_RS03805	ABC transporter permease	4.4E-139	-6.40246917
36	LBJ_RS08115	hypothetical protein	8.5E-137	-6.14272582
37	LBJ_RS08140	cbb3-type cytochrome oxidase assembly protein CcoS	1.4E-135	-10.6361402
38	LBJ_RS15055	response regulator	4.4E-134	-4.88366073
39	LBJ_RS11175	hypothetical protein	1.5E-132	40.91118591
40	LBJ_RS12580	putative lipoprotein	6.5E-130	-4.57465474
41	LBJ_RS12800	TetR/AcrR family transcriptional regulator	6E-128	-6.67402592
42	LBJ_RS15535	LIC_13355 family lipoprotein	2.6E-123	-3.49449646
43	LBJ_RS02690	protein-glutamate O-methyltransferase CheR	7.1E-123	-4.03909246
44	LBJ_RS12430	bile acid:sodium symporter family protein	1E-122	5.970576464
45	LBJ_RS16085	IS110 family transposase	1.9E-119	60.86820245
46	LBJ_RS03450	membrane protein	3E-118	-24.2667367
47	LBJ_RS09490	sulfate ABC transporter substrate-binding protein	3.2E-118	-3.79141212
48	LBJ_RS03565	DUF2339 domain-containing protein	5.7E-118	-3.16637168
49	LBJ_RS15290	CHAT domain-containing protein	9.4E-118	-4.6868882
50	LBJ_RS13795	hypothetical protein	2E-115	7.424443586

**Table 3:** The fifty most significantly differentially expressed genes between JB197 vs. HB203 grown at 37°C. All genes are relative to the JB197 reference genome and annotated by the JB197 gene ID. *Positive fold change values denote higher transcription in JB197 and conversely, negative values indicate higher expression in HB203.

No.	Gene ID	Description	Adjusted p-value	Fold Change*
1	LBJ_RS14715	thiol peroxidase	0	-13.1190795
2	LBJ_RS11060	hypothetical protein	3.5296E-302	-54.5863068
3	LBJ_RS10820	IS110 family transposase	6.5137E-257	-35.1194343
4	LBJ_RS11760	AMP-binding protein	3.3684E-231	62.22334802
5	LBJ_RS13430	hypothetical protein	1.363E-230	629.2203707
6	LBJ_RS03805	ABC transporter permease	8.3752E-219	-6.48077195
7	LBJ_RS20375	Pseudogene	1.7778E-206	733.8727978
8	LBJ_RS01625	hypothetical protein	2.4896E-198	-16.1431113
9	LBJ_RS12800	TetR/AcrR family transcriptional regulator	1.9525E-178	-10.9824144
10	LBJ_RS15545	nicotinate-nucleotide—dimethylbenzimidazole phosphoribosyltransferase	1.6142E-171	-4.56744637
11	LBJ_RS12430	bile acid:sodium symporter family protein	3.2135E-155	7.258551937
12	LBJ_RS04775	universal stress protein	1.1582E-152	-11.0326908
13	LBJ_RS16210	DUF1304 domain-containing protein	1.4728E-151	-4.45075523
14	LBJ_RS18695	universal stress protein	7.068E-140	-13.5862528
15	LBJ_RS03800	SDR family NAD(P)-dependent oxidoreductase	2.7028E-139	-5.36080725
16	LBJ_RS15535	LIC_13355 family lipoprotein	1.2843E-133	-4.07222015
17	LBJ_RS14495	IS110 family transposase	5.5607E-123	12.73016459
18	LBJ_RS10950	DUF1761 domain-containing protein	1.4478E-121	-6.86295759
19	LBJ_RS11765	hypothetical protein	3.5124E-104	297.1375462
20	LBJ_RS01235	alginate export family protein	2.9715E-102	-11.0853794
21	LBJ_RS11055	sensor histidine kinase	1.33311E-98	-3.74558988
22	LBJ_RS06350	IS110 family transposase	1.65155E-98	307.0006006
23	LBJ_RS01505	hypothetical protein	8.73598E-98	-6.31596704
24	LBJ_RS12795	2-isopropylmalate synthase	5.80332E-97	3.433367106
25	LBJ_RS15540	hypothetical protein	2.21913E-93	-5.29469227
26	LBJ_RS19155	transposase	2.96544E-92	-6.27039105
27	LBJ_RS01970	TonB-dependent receptor plug domain-containing protein	5.05976E-91	-3.47969671
28	LBJ_RS18335	helicase	2.03011E-87	-4.8292601
29	LBJ_RS00400	hypothetical protein	3.25127E-86	-3.19910647
30	LBJ_RS15060	HAMP domain-containing histidine kinase	6.53107E-86	-4.90567874
31	LBJ_RS03235	DUF1561 domain-containing protein	8.31078E-80	4.024464576
32	LBJ_RS16085	IS110 family transposase	7.76962E-78	67.75325409
33	LBJ_RS14360	PilZ domain-containing protein	4.4611E-75	-3.3401493
34	LBJ_RS10080	IS3 family transposase	1.9871E-74	-4.36805694
35	LBJ_RS15770	IS110 family transposase	1.33564E-69	10.77676664
36	LBJ_RS13725	DUF1566 domain-containing protein	1.52252E-68	-4.0367724
37	LBJ_RS13365	adenylate/guanylate cyclase domain-containing protein	1.74938E-65	22.24371838
38	LBJ_RS07510	hypothetical protein	1.78604E-65	-25.6351281
39	LBJ_RS12775	phage holin family protein	4.48069E-64	-3.53860296
40	LBJ_RS17005	IS110 family transposase	3.16703E-63	-5.07502335
41	LBJ_RS08330	hypothetical protein	1.62373E-62	-3.56562162
42	LBJ_RS15105	fatty acid desaturase	1.82837E-61	-5.34252404
43	LBJ_RS12410	alpha/beta hydrolase	1.17454E-60	3.729386778
44	LBJ_RS03005	methyl-accepting chemotaxis protein	1.45254E-55	-3.80909227
45	LBJ_RS20730	hypothetical protein	9.86244E-55	-3.0177925
46	LBJ_RS05375	hypothetical protein	3.02277E-53	-8.16495142
47	LBJ_RS02135	sensor domain-containing diguanylate cyclase	5.7787E-53	3.37224051
48	LBJ_RS15530	hypothetical protein	5.35322E-52	-3.43469974
49	LBJ_RS19470	hypothetical protein	6.28568E-52	-9.60546819
50	LBJ_RS17065	tetratricopeptide repeat protein	2.43878E-50	-5.14403032

**Table 4:** The fifty most significantly differentially expressed genes between JB197 grown at 37°C and 29°C. All genes are relative to the JB197 reference genome and annotated by the JB197 gene ID. *Positive and negative fold change values indicate higher expression at 37 and 29°C, respectively.

No.	Gene ID	Description	Adjusted p-value	Fold Change*
1	LBJ_RS19915	hypothetical protein	4.1E-211	-26.18877429
2	LBJ_RS14945	hypothetical protein	3.7E-199	-26.36202418
3	LBJ_RS02895	hypothetical protein	1.2E-154	-37.40296337
4	LBJ_RS13800	DUF736 family protein	2.3E-127	-10.01744691
5	LBJ_RS15950	hypothetical protein	8E-123	-32.92221868
6	LBJ_RS06880	nuclear transport factor 2 family protein	1.2E-104	5.687822012
7	LBJ_RS02880	sigma-70 family RNA polymerase sigma factor	4.46E-98	-11.4412595
8	LBJ_RS00360	hypothetical protein	7.71E-94	-8.995650267
9	LBJ_RS11175	hypothetical protein	8.43E-89	-23.46187329
10	LBJ_RS13795	hypothetical protein	1.5E-75	-4.507308623
11	LBJ_RS14935	penicillin acylase family protein	4.96E-74	-4.263602348
12	LBJ_RS06790	hypothetical protein	4.28E-70	-7.21434451
13	LBJ_RS05715	ABC transporter ATP-binding protein	9.54E-69	-3.057019984
14	LBJ_RS02875	hypothetical protein	1.21E-66	-9.45213817
15	LBJ_RS19620	LemA domain protein	5.13E-59	-4.570885767
16	LBJ_RS05665	N-6 DNA methylase	1.94E-56	-3.279854045
17	LBJ_RS16215	hypothetical protein	2.89E-55	-4.296096957
18	LBJ_RS01605	sphingomyelinase C	5.14E-55	-3.140044775
19	LBJ_RS03440	hypothetical protein	1.49E-49	33.15690713
20	LBJ_RS01595	hypothetical protein	2.36E-46	-3.441367833
21	LBJ_RS14170	GNAT family N-acetyltransferase	9.62E-46	-4.015319302
22	LBJ_RS13105	c-type cytochrome	1.09E-45	-3.571787134
23	LBJ_RS08105	cytochrome-c oxidase%2C cbb3-type subunit I	3.61E-45	3.603606487
24	LBJ_RS08030	chromosome segregation protein SMC	3.49E-44	3.006011374
25	LBJ_RS14995	hypothetical protein	5.6E-44	-3.306536499
26	LBJ_RS10860	transcriptional repressor LexA	1.68E-43	-3.282708081
27	LBJ_RS12935	imelysin family protein	1.05E-40	3.390817097
28	LBJ_RS00735	ACT domain-containing protein	1.19E-40	-3.224212388
29	LBJ_RS20620	hypothetical protein	1.21E-40	-4.299539877
30	LBJ_RS20080	hypothetical protein	1.14E-39	-4.777687914
31	LBJ_RS03510	dihydrofolate reductase family protein	6.59E-39	-3.097280537
32	LBJ_RS13355	Rpn family recombination-promoting nuclease/putative transposase	7.45E-39	-3.26226348
33	LBJ_RS08110	cbb3-type cytochrome c oxidase subunit II	1.35E-38	3.055046955
34	LBJ_RS08355	glycosyltransferase	2.36E-38	3.89582743
35	LBJ_RS17015	IS3 family transposase	3.22E-38	-3.164228522
36	LBJ_RS19535	IS110 family transposase	6.53E-38	-5.057228585
37	LBJ_RS13885	hypothetical protein	9.93E-38	-3.10221434
38	LBJ_RS05295	DNA repair protein RadC	6.55E-36	-3.18213023
39	LBJ_RS02000	STAS domain-containing protein	1.63E-35	-3.086491683
40	LBJ_RS00270	IS110 family transposase	1.88E-34	-3.472082534
41	LBJ_RS13100	methylamine utilization protein	4.19E-34	-3.498664369
42	LBJ_RS03940	carboxylate—amine ligase	5.26E-34	-3.31000339
43	LBJ_RS03730	tyrosine-type recombinase/integrase	1.87E-33	-3.712961519
44	LBJ_RS00275	IS110 family transposase	2.37E-33	-3.806618894
45	LBJ_RS14180	hypothetical protein	1.12E-32	-4.207651574
46	LBJ_RS05670	bacterioferritin	2.12E-32	-3.11119954
47	LBJ_RS20470	IS110 family transposase	4.65E-32	-12.55137177
48	LBJ_RS05835	flagellar basal body M-ring protein FliF	3.3E-31	4.219471408
49	LBJ_RS09455	hypothetical protein	4.84E-31	-3.272227444
50	LBJ_RS16285	hypothetical protein	1.05E-30	-4.593857724

**Table 5:** The forty-one significantly differentially expressed genes between HB203 grown at 29°C and 37°C. All genes are relative to the HB203 reference genome and annotated by the HB203 gene ID. *Positive and negative fold change values indicate higher expression at 37 and 29°C, respectively.

No.	Gene ID	Description	Adjusted p-value	Fold Change*
1	B9T54_RS07815	ferredoxin	1.33546E-87	3.517131533
2	B9T54_RS11395	hypothetical protein	5.19401E-82	3.708465663
3	B9T54_RS17825	hypothetical protein	3.61907E-79	-4.120692145
4	B9T54_RS14810	N-6 DNA methylase	3.71389E-67	3.254218463
5	B9T54_RS03260	hypothetical protein	2.87504E-66	-3.447448992
6	B9T54_RS15150	heat-inducible transcription repressor HrcA	1.05609E-64	6.533979347
7	B9T54_RS01900	dienelactone hydrolase	1.5023E-61	4.658423574
8	B9T54_RS07620	hypothetical protein	1.1521E-60	-10.51341747
9	B9T54_RS03575	type I-E CRISPR-associated endonuclease Cas1	1.73751E-58	-6.378037244
10	B9T54_RS08850	lipoprotein LipL45	1.96136E-58	4.530223455
11	B9T54_RS07625	cell envelope integrity protein CreD	8.83948E-47	-5.288740461
12	B9T54_RS14025	hypothetical protein	1.77149E-46	-3.090558069
13	B9T54_RS04800	response regulator	2.49181E-45	-3.163422499
14	B9T54_RS15550	hypothetical protein	5.91582E-40	-3.473303521
15	B9T54_RS17060	hypothetical protein	9.60714E-35	3.187895636
16	B9T54_RS03570	type I-E CRISPR-associated endoribonuclease Cas2	3.45695E-33	-7.028343167
17	B9T54_RS11680		4.34635E-33	-3.235679043
18	B9T54_RS06710	DMT family protein	9.86399E-32	5.820718943
19	B9T54_RS07655	hypothetical protein	3.1673E-31	-4.606051717
20	B9T54_RS15880	hypothetical protein	6.85616E-29	-3.038741126
21	B9T54_RS02250	pirin family protein	6.96224E-29	4.453810909
22	B9T54_RS15140	molecular chaperone DnaK	4.79434E-26	3.367846993
23	B9T54_RS03255		1.34471E-25	-4.505460396
24	B9T54_RS14795	RNA-binding protein	3.49859E-25	-3.015954587
25	B9T54_RS02245		2.77228E-23	4.078842911
26	B9T54_RS14190	heavy-metal-associated domain-containing protein	3.77156E-17	3.210076413
27	B9T54_RS14055	hypothetical protein	2.17824E-14	-3.143301857
28	B9T54_RS14040	hypothetical protein	7.7841E-14	-5.092670388
29	B9T54_RS15145	nucleotide exchange factor GrpE	2.95709E-12	4.627117767
30	B9T54_RS06715	Hsp20/alpha crystallin family protein	3.50722E-11	12.13060651
31	B9T54_RS02240	IS110 family transposase	4.35816E-11	3.359078032
32	B9T54_RS04890	hypothetical protein	1.62888E-10	3.352647477
33	B9T54_RS14065	LigB lipoprotein	6.80261E-08	-3.857388898
34	B9T54_RS06720	Hsp20/alpha crystallin family protein	8.03618E-08	11.29627595
35	B9T54_RS12110	hypothetical protein	1.91955E-07	5.779451745
36	B9T54_RS13435	hypothetical protein	5.45037E-07	3.141132189
37	B9T54_RS06340	hypothetical protein	1.15478E-05	3.656406252
38	B9T54_RS01250		1.53061E-05	3.793771781
39	B9T54_RS10380	ATP-dependent chaperone ClpB	2.28633E-05	3.770143794
40	B9T54_RS05615	hypothetical protein	7.52809E-05	3.651943751
41	B9T54_RS14145	hypothetical protein	0.005394822	3.219921943

**Fig 4:**
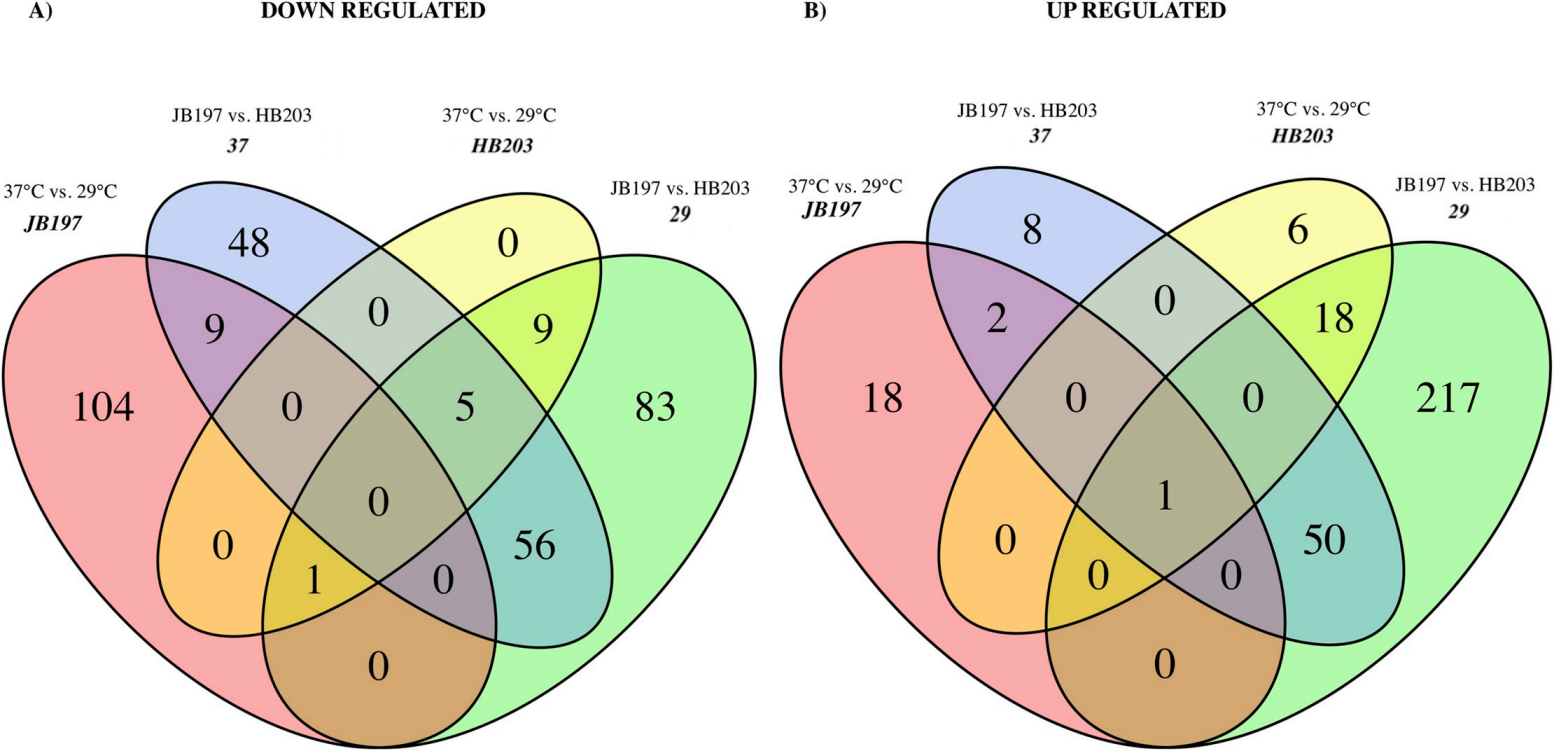
Venn diagram of differential expression gene profiles among the four primary contrasts of interest (label in italics); JB197 (37°C vs. 29°C, pink), HB203 (37°C vs. 29°C, yellow), 29°C (JB197 vs. HB203, green), and 37°C (JB197 vs. HB203, blue). Shown in panel (A) are the significantly down regulated DE genes, and (B) denotes the significantly up regulated DE genes.

To identify DE genes conserved across comparisons, Venn diagrams were constructed amongst the four contrasts of interest, **Fig 4**. Venn diagrams were created separately for ‘down’ regulated (**Fig 4A**) and ‘up’ regulated (**Fig 4B**) DE genes. Surprisingly, very little overlap exists between the DE gene profiles between contrasts. No genes were identified as being shared between all contrasts in the down regulated/negative fold change diagram (**Fig 4A**), and the lone gene (LBJ_RS13430, currently annotated as YP_801646.1) that was identified in all four contrasts of the up regulated/positive fold change diagram (**Fig 4B**) encodes a hypothetical protein with no predicted function or conserved motifs. The most conserved gene groups appear between 29°C and 37°C contrasts, with 61 genes being down regulated in JB197 compared to HB203 at 29°C and 37°C (blue and green circle overlap **Fig 4A**), and 51 genes being up regulated in JB197 compared to HB203 in 29°C and 37°C (blue and green circle overlap **Fig 4B**). The 61 shared genes down regulated in JB197 within both temperatures include several lipoproteins (LBJ_RS19300, LIC_13355 family lipoprotein, LBJ_RS15535), LigB (LBJ_RS03415), a universal stress protein (LBJ_RS18695), and genes predicted to encode hypothetical proteins. Of the 51 genes up regulated in JB197 compared to HB203 at both temperatures, many were transposases of either the IS110 or ISF5 family, several transport proteins (MMPL family transporter LBJ_RS12280), and efflux RND transporter permease subunit (LBJ_RS03290)), as well as similar to the down regulated series, genes encoding hypothetical proteins.

Of the significantly DE genes identified between HB203 and JB197 cultured at 29°C, approximately 124 (28.2%) are annotated as encoding hypothetical proteins; at 37°C, there are 49 (27.4%), **S2 and S3 Tables**. Additional annotated DE genes include those involved in facilitating transmembrane transport and signaling, specifically, heavy metal translocating P-type ATPase, ABC transporters, and several response regulators. Well characterized outer membrane proteins were also differentially expressed including *lipL32* (LBJ_RS09045) and *lipL41* (LBJ_RS01765) at 29°C (adj. p-value = 2.5E-70, FC = -6.0 and adj. p-value = 2.3E-37, FC = -3.3 respectively), all of which are expressed less in JB197 compared to HB203 **S2 Table**.

Similarly, comparison of JB197 cultured at 37 vs. 29°C identified 44 (32.6%) of the most significantly DE genes as encoding hypothetical proteins while 16 (38%) were identified in the 41 DE genes comparing HB203 at 37 and 29°C, **Tables 4 and 5**. Of note, DE genes in JB197 at 37 and 29°C include *lipL32* (LBJ_RS09045, adj. p-value = 8.4E-28, FC = 3.3), iron storage protein bacterioferritin (LBJ_RS05670, adj. p-value = 2.1E-32, FC = -3.1), the enzyme sphingomyelinase C (LBJ_RS01605, adj. p-value = 5.1E-55, FC = -3.1), and numerous genes related to flagellin/flagellar bodies (LBJ_RS05835 and LBJ_RS06930 which increased in expression at 37°C, and LBJ_RS10715 which has a decreased expression), **S4 Table**. DE genes in HB203 at 37 vs. 29°C include *ligB* (adj. p-value = 6.8E-8, FC = -3.9), *lipL45* (LBJ_RS07310/ B9T54_RS08850, adj. p-value = 2.0E-58, FC = 4.5), and negative regulator of heat shock proteins heat-inducible transcription repressor HrcA (LBJ_RS02385/ B9T54_RS15150, adj. p-value = 1.1E-64, FC = 6.5), **Table 5**.

### Most highly expressed genes

The most highly expressed genes and annotated sRNAs by normalized read count, in both JB197 and HB203, at both 37 and 29°C, include tmRNA, RNase P, dnaK, groEL, Hsp20, elongation factor Tu, elongation factor G, DNA-directed RNA polymerase subunits, flagellin, as well as several genes encoding outer membrane proteins including *lipL32*, *lipL21* and *lipL41*. Additionally, genes encoding hypothetical proteins also consistently appear as highly expressed across contrasts, including LBJ_RS01915 (HB203 gene ID B9T54_RS15650) and LBJ_RS04980 (HB203 gene ID B9T54_RS05120) (**S2–S5 Tables**).

### LigB is lowly expressed by JB197 compared to HB203

**Fig 5:**
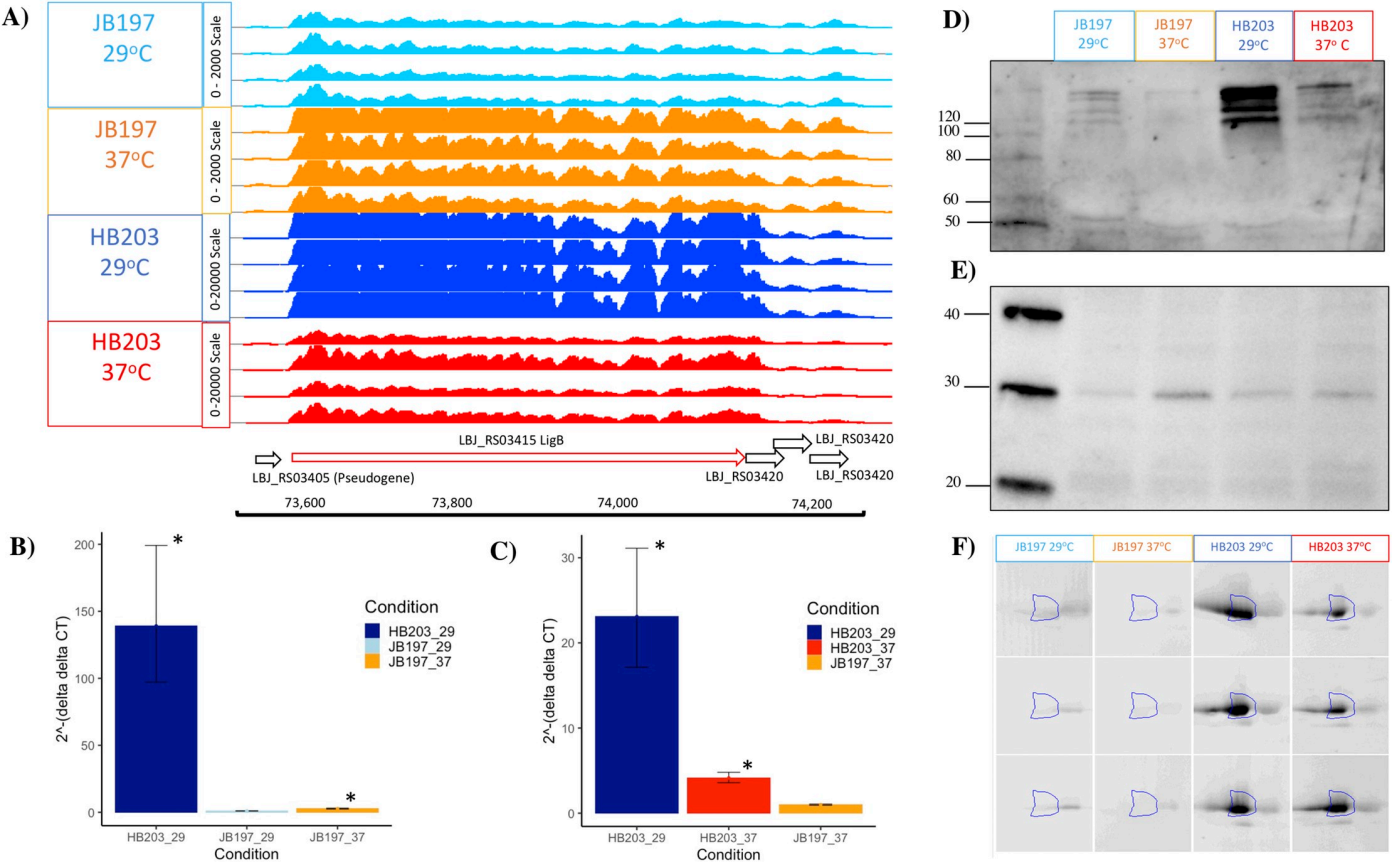
Evaluation of LigB expression in JB197 and HB203. (A) Integrated Genome Browser (IGB) view of gene expression (light blue = JB197 at 29°C, orange = JB197 at 37°C, dark blue = HB203 at 29°C, red = HB203 at 37°C). Note that Y-axis normalized read count scales are different; JB197 (both temperatures) was scaled to 0–2,000, while HB203 (both temperatures) was scaled 0–20,000. Validation of RNAseq data by RT-qPCR of *ligB* relative to JB197 at 29°C, using *secA* as the control gene for the 29°C (JB197 vs. HB203) and JB197 (37°C vs. 29°C) contrasts (B) or *rho* as the control gene for the 37°C (JB197 vs. HB203) and HB203 (37°C vs. 29°C) contrasts normalized to the expression of JB197 37°C (C). Each lane of the immunoblot contains approximately 5 x 10^8^ leptospires of *L*. *borgpetersenii* serovar Hardjo strain JB197 and HB203 cultured at 29 or 37°C with (D) anti-LigB and (E) anti-LipL45. (F) DIGE of LigB in strain JB197 and HB203 cultured at 29 or 37°C. Molecular mass markers (kDa) are indicated. * indicates a p-value < 0.05. Error bars represent 95% confidence intervals.

Notably, the well characterized outer membrane lipoprotein LigB encoding gene was identified as highly differentially expressed between the two strains (lower in JB197 compared to HB203), whether cultured at 29°C (adj. p-value = 8.9E-278, FC = -54.1) or 37°C (adj. p-value = 6.1E-10, FC = -6.1) (**Tables 2 and S2**). Within JB197, *ligB* was significantly more highly expressed at 37°C compared to 29°C (adj. p-value = 7.0E-7, FC = 2.3), (**S4 Table**). In contrast, *ligB* was significantly more highly expressed by HB203 at 29°C compared to 37°C (adj. p-value = 6.8E-8, FC = -3.9), **Table 5**. Given the role of LigB as a well characterized virulence factor, as well as its reported ability to act as a protective vaccinogen of pathogenic leptospires, the expression of *ligB* was investigated further, **Fig 5**. Visualization of the RNAseq data by IGB confirms the large numbers of transcripts for *ligB* in HB203, at both 29 and 37°C, compared to that of JB197 (**Fig 5A)** (note the difference in read count scales). Differential expression of *ligB* was confirmed by RT-qPCR by comparison with two different control genes. Since common bacterial control genes varied in levels of expression across contrasts, *secA* was used as the control gene for the 29°C (JB197 vs. HB203) and JB197 (37°C vs. 29°C) contrasts (**Fig 5B**), and *rho* was used as the control gene for the 37°C (JB197 vs. HB203) and HB203 (37°C vs. 29°C) contrasts (**Fig 5C**). In addition, immunoblotting of whole cell sonicates with antibody specific for LigB confirms that HB203 expresses much larger amounts of LigB compared to that of JB197 (**Fig 5D)**. In agreement with RNAseq data, detection of LigB by immunoblotting is diminished in HB203 when cultured at 37 compared to 29°C. However, in contrast to RNAseq data which indicates that expression of LigB in JB197 is increased when cultures are maintained at 37°C compared to 29°C, the opposite was apparent by immunoblotting and less LigB was detected in JB197 maintained at 37°C compared to 29°C. As an additional control to demonstrate contrasting differential expression of antigens, immunoblots were also performed with anti-LipL45, an outer membrane lipoprotein originally annotated as Qlp42 [37, 38] which is increased in expression in *L*. *interrogans* cultured at 37°C compared to 29°C, and identified as a gene that was significantly DE by RNAseq in HB203 at 37°C compared to 29°C (adj. p-value = 1.96E-58, FC = 4.5) (**Fig 5D** and **Table 5**). To further investigate and validate expression of LigB as a component of the serovar Hardjo outer membrane, fractions enriched for the outer membrane proteome of HB203 were compared to that of JB197 by 2-D DIGE; a protein spot identified as LigB by mass spectrometry was detected in six biological replicates of HB203 cultured at 29 or 37°C in larger amounts compared to that of six biological replicates of JB197 (p-value = 2.9E-07, q = 6.3E-07, Power ≥ 0.999, FC = 28.7) (**Fig 5F**). The identification of LigB by mass spectrometry was based on the identification of 26 unique peptides and 110 Peptide Spectrum Match (PSMs) (sequence coverage of this 1896 amino acid protein was 16%).

In addition to *ligB*, qPCR was used to validate expression of *lipL45* across all four major contrasts of interest (see above) (**S3 Fig**). Validation of gene expression by qPCR is also provided for LBJ_RS02895 and LBJ_RS11060, (**S4 Fig**); LBJ_RS02895, which encodes a hypothetical protein, was significantly differentially expressed in both 29°C (JB197 vs. HB203) and JB197 (37°C vs. 29°C) contrasts (adj. p-value = 0.0, FC = 220.5, adj. p-value = 1.2E-154, FC = -37.4 respectively). LBJ_RS11060, which also encodes a hypothetical protein, was significantly differentially expressed in the 37°C (JB197 vs. HB203) contrast (adj. p-value = 3.5E-302, FC = -54.6).

### Differential expression of lipopolysaccharide and genes within the *rfb* locus

**Fig 6:**
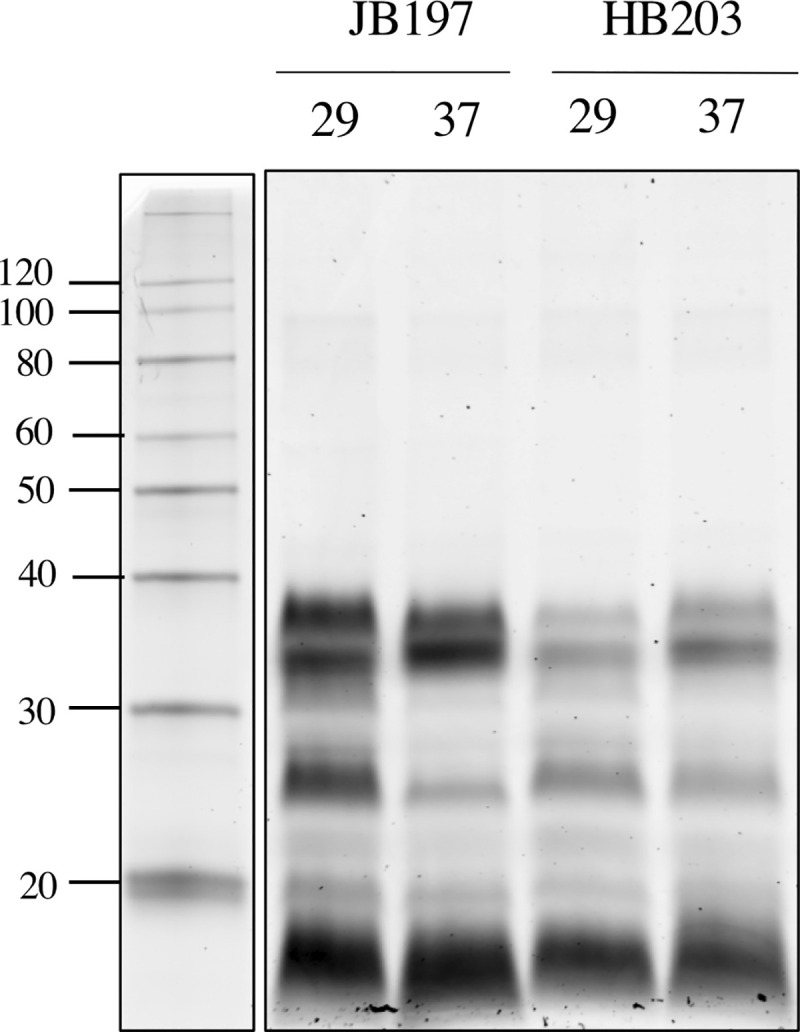
Evaluation of lipopolysaccharide expression in JB197 and HB203. Total lipopolysaccharide expressed by the equivalent of 5ug of serovar Hardjo strain JB197 and strain HB203 at 29 or 37°C. Molecular mass markers (kDa) are indicated.

LPS of pathogenic leptospires is defined according to serovar status and is considered a protective antigen against homologous challenge. Inclusion of specific serovars within bacterin vaccines are believed to mediate protection via LPS. Given the importance of individual strains of *Leptospira* as components of bacterin vaccines to mediate protection against other strains of the same serovar, the expression of LPS by serovar Hardjo strains JB197 and HB203 was investigated further. Interestingly, JB197 produced larger amounts of LPS compared to that of HB203 when visualized after separation by gel electrophoresis (**Fig 6**). Accordingly, genes within the *rfb* locus, as defined by GenBank Accession AF078135.1 [18], were examined more closely to identify DE genes. No genes within the *rfb* locus met the DE criteria of significance and fold change of three. However, when fold change criterion was lowered to two, nine genes were DE between JB197 and HB203 cultured at 29°C (**Table 6**). Surprisingly, all nine were more highly expressed in HB203 compared to JB197. At 37°C, no genes were DE with a two-fold significance threshold, although LBJ_RS06485 annotated as a methyltransferase, was DE at 1.95 fold (adj. p-value = 5.6E-09) and present in higher amounts in HB203 compared to JB197.

**Table 6:** Differential expression examination of genes within the *rfb* locus as well as outside the *rfb* locus but annotated as pertaining to LPS. Differentially expressed genes with an adjusted p-value < 0.05, and fold change minimum of two are shaded in gray.

Gene ID	Annotation	29°C (JB197 vs. HB203)		37°C (JB197 vs. HB203)		JB197 (37°C vs. 29°C)		HB203 (37°C vs. 29°C)	
		Adj. p-value	Fold Change	Adj. p-value	Fold Change	Adj. p-value	Fold Change	Adj. p-value	Fold Change
**Genes contained in the *rfb* region**									
LBJ_RS06390	glycosyltransferase	1.64E-10	-1.86	3.11E-3	-1.26	3.26E-4	1.40	0.42	-1.07
LBJ_RS06395	N-acetyl sugar amidotransferase	3.56E-16	-1.57	0.97	-1.00	3.42E-8	1.40	3.58E-3	-1.14
LBJ_RS06400	imidazole glycerol phosphate synthase subunit HisH	3.84E-25	-1.80	0.70	1.03	9.0E-8	1.42	4.92E-7	-1.33
LBJ_RS06405	imidazole glycerol phosphate synthase subunit HisF	1.68E-16	-1.78	0.54	1.04	4.13E-7	1.38	7.15E-6	-1.37
LBJ_RS06410	glycosyltransferase family 4 protein	2.68E-18	-2.16	0.05	-1.15	2.30E-6	1.56	3.59E-3	-1.23
LBJ_RS06415	SDR family NAD(P)-dependent oxidoreductase	1.72E-8	-1.61	0.03	1.16	3.82E-4	1.37	6.11E-6	-1.39
LBJ_RS06420	NTP transferase domain-containing protein	7.07E-17	-2.01	0.51	1.05	8.53E-7	1.57	5.55E-7	-1.37
LBJ_RS06425	NAD(P)-dependent oxidoreductase	8.74E-31	-1.93	1.0	-1.00	1.99E-10	1.52	2.22E-9	-1.30
LBJ_RS06430	SDR family oxidoreductase	6.60E-29	-2.15	0.24	-1.06	3.05E-12	1.59	4.40E-7	-1.31
LBJ_RS06435	polysaccharide biosynthesis protein	2.53E-21	-1.93	0.97	-1.00	7.85E-11	1.68	1.00E-3	-1.17
LBJ_RS06440	UDP-N-acetylglucosamine 2-epimerase (non-hydrolyzing)	2.99E-26	-2.20	0.02	-1.10	1.10E-15	1.72	3.92E-3	-1.19
LBJ_RS06445	glycosyltransferase family 4 protein	1.26E-27	-2.52	2.20E-3	-1.22	3.44E-12	1.92	0.14	-1.09
LBJ_RS06450	exopolysaccharide biosynthesis polyprenyl glycosylphosphotransferase	3.02E-18	-2.07	0.64	-1.04	5.38E-11	1.83	0.13	-1.11
LBJ_RS06455	oligosaccharide repeat unit polymerase	1.81E-5	-1.82	0.29	-1.15	4.20E-10	2.02	0.17	1.25
LBJ_RS06475	flippase	7.11E-12	-2.08	7.11E-4	-1.42	1.59E-4	1.54	0.86	1.03
LBJ_RS06480	glycosyltransferase family 2 protein	1.01E-12	-1.96	2.20E-3	-1.31	6.53E-5	1.52	0.25	-1.01
LBJ_RS06485	methyltransferase%2C TIGR04325 family	1.51E-15	-2.33	5.62E-9	-1.95	0.17	1.20	2.17E-3	-1.01
LBJ_RS06490	glycosyl transferase	0.01	-1.37	0.04	-1.23	2.99E-3	1.32	0.85	1.17
LBJ_RS06495	WxcM-like domain-containing protein	0.01	-1.41	0.01	-1.34	0.48	1.09	0.90	1.02
LBJ_RS06500	DegT/DnrJ/EryC1/StrS family aminotransferase	1.46E-10	-1.60	0.01	-1.22	1.08E-3	1.30	0.87	-1.03
LBJ_RS06505	glycosyltransferase	8.68E-5	-1.45	2.71E-13	-1.69	3.93E-3	1.28	0.25	1.46
LBJ_RS06510	glycosyltransferase	3.38E-7	-1.64	1.41E-6	-1.50	0.40	1.10	0.95	-1.02
LBJ_RS06520	glycosyltransferase	5.66E-9	-1.72	8.46E-5	-1.40	0.03	1.25	0.61	1.00
LBJ_RS06525	dTDP-4-dehydrorhamnose 3%2C5-epimerase	7.92E-11	-1.74	1.83E-3	-1.21	1.31E-4	1.37	1.66E-5	-1.07
LBJ_RS06530	dTDP-4-dehydrorhamnose reductase	1.34E-22	-2.08	1.13E-7	-1.51	0.02	1.25	0.80	-1.12
LBJ_RS06535	dTDP-glucose 4%2C6-dehydratase	4.54E-18	-1.91	0.50	-1.06	0.02	1.28	0.81	-1.44
LBJ_RS06540	glucose-1-phosphate thymidylyltransferase RfbA	1.57E-8	-1.74	0.96	-1.01	0.04	1.32	0.95	-1.33
**Genes not contained in the *rfb* region but pertaining to LPS**									
LBJ_RS07965	LPS export ABC transporter ATP-binding protein	0.03	-1.17	0.99	1.00	0.50	-1.07	5.56E-3	-1.29
LBJ_RS07975	LPS export ABC transporter periplasmic protein LptC	4.63E-3	-1.27	1.00	1.00	0.66	1.06	0.06	-1.23
LBJ_RS08415	O-antigen ligase family protein	4.95E-15	-1.72	1.29E-4	-1.32	1.39E-05	1.42	0.41	1.06
LBJ_RS09125	LPS-assembly protein LptD	0.01	-1.16	8.01E-9	1.50	0.09	1.14	2.66E-16	-1.57

Closer inspection of the *rfb* locus with IGB identified non-coding expression in the flanking intergenic regions of select genes within the *rfb* loci (**S5 Fig**).

Additional genes not contained within the *rfb* locus, but annotated as having a function related to LPS were identified; these included LBJ_RS07965 (annotated as encoding an LPS export ABC transporter ATP-binding protein), LBJ_RS07975 (annotated as encoding the LPS export ABC transported periplasmic protein LptC protein), LBJ_RS08415 (annotated as encoding an O-antigen ligase family protein) and LBJ_RS09125 (annotated as encoding the LPS-assembly protein LptD). Although several of these genes met adjusted p-value thresholds for significance, none were significant by either fold change three or fold change two standard (see **Table 6**).

## Discussion

While the genomes of *L*. *borgpetersenii* serovar Hardjo strains JB197 and HB203 are strikingly similar at the nucleotide level and the serovar identity identical (both type Hardjo bovis), the disease phenotypes produced in a hamster infection model are drastically different. This suggests that variation at the strain level of *Leptospira* classification plays an important role in host response and species-specific interactions between host and pathogen. Interestingly, a genomic comparison identified that *L*. *borgpetersenii* is ~700 kb smaller than *L*. *interrogans* and was hypothesized to be undergoing insertion sequence mediated genome reduction, with evolution towards a strict-host-host transmission cycle and loss of gene function centered on impairment of environmental sensing and metabolite transport and utilization [22]. The characterization of the *L*. *borgpetersenii* transcriptome is an important asset in assessing conserved and unique factors of infection across strains and serovars as well as comparing evolutionary changes across divergent species of *Leptospira*. The unique species-specific attributes of leptospiral interactions (both at the host and pathogen level), along with their sensitive response to the environment are major contributing factors heavily associated with persistent disease transmission. Understanding the sensitivity and specificity of *Leptospira* at the strain level is critical to the development of the next generation of therapeutics and ultimately is required for disease control.

When examining the genes most highly expressed across conditions, many are identical to the top 50 most expressed genes previously identified for *L*. *interrogans* serovar Copenhageni strain L1-130 when cultured at either 29°C, or within dialysis membrane chambers implanted in the peritoneal cavity of rats. Among these highly expressed genes were *ligB*, *lipL32*, *lipL21* and *lipL41*, despite the different growth environments offered by EMJH versus HAN media, as well as significant genomic differences [39]. This suggests a level of consistency of base leptospiral mechanics across pathogenic species and across vastly different environments. A base level of conserved transcriptomic activity also helps emphasize strain specific nuances that may play an important role in *Leptospira* and host interactions, severity of disease, and immune response and/or escape. Also of interest, this study highlights several genes encoding hypothetical proteins that are conserved across contrasts of interest. For example, LBJ_RS01915 is a gene encoding a hypothetical protein that is in the top fifty most highly expressed genes in all of the major contrasts of interest (see **S2–S5 Tables**). Similarly, both genes LBJ_RS11060 (HB203 B9T54_RS15165) and LBJ_RS01505 (HB203 B9T54_RS10450) encode hypothetical proteins that are significantly DE in both 29°C and 37°C contrasts comparing the two strains and both genes are more highly expressed in HB203 compared to JB197 at both temperatures (see **S2 and S3 Tables**). These hypothetical proteins warrant further characterization which is now being facilitated via the use of HAN media allowing culture maintenance at 37°C and the continued development of *Leptospira* mutagenesis [40, 41]. This is also further evidence that while leptospirosis research has focused on pan-genome genetic typing of *Leptospira* species, a ‘pan-transcriptome’ approach may offer novel insights to serovar or species-specific behavior.

The divergent phenotypes seen in the hamster model after experimental challenge and the differences in total protein composition seen in **Fig 1** suggested additional investigation of strain specific profiles at the transcriptomic level could be insightful. We investigated the differential gene expression between JB197 and HB203 cultured at 37°C and 29°C. As illustrated by the PCA analysis (**Fig 2**) and supported by heatmap visualization (**Fig 3**), the data shows biological replicates clustered more tightly together due to strain than due to temperature. Although, HB203 at 37°C and 29°C cluster more tightly than JB197 at 37°C and 29°C, which suggested greater gene expression differences within JB197 between temperatures than in HB203. The high number of DE genes in the JB197 contrast (135 genes between 37°C and 29°C) compared to only 41 DE genes for the HB203 contrast (37°C and 29°C) further supports this observation. Interestingly the highest levels of differential gene expression are seen from the 29°C contrast (JB197 vs. HB203), both visually by heatmap (**Fig 3**) and by number of DE genes (440 compared to 197 for the 37°C contrast between strains). This is a particularly critical result for several reasons. First, this is the temperature at which all *in vitro* culture of *Leptospira* have traditionally been maintained, which has vast implications for the differential behavior of different strains, especially those being cultured for bacterin vaccine use. Second, this more closely resembles the temperature of ex-host environment natural leptospires may encounter in the wild. The characterization of these gene expression changes is important for illuminating changes leptospires go through as they travel from host to environment to host. Changes occurring at these transition points may point to targets for the development of prevention technologies or environmental clean-up and control. The high levels of DE seen in the 29°C contrast were not seen with the strain comparisons at 37°C, suggesting a possible maintenance or homeostasis of the pathogen at an incubated temperature similar to what a host kidney/tissue environment would look like. This is also interesting in the context of colonization of leptospires in different tissues and the interaction with the host immune system that can result in clearance of the pathogen, chronic colonization of the host kidney, or severe multi-organ infection depending on the challenging *Leptospira*. Further, interesting among highly DE genes was the lack of conservation of DE genes across all conditions. While the most conserved genes were found in the comparisons between strains at 29°C and 37°C, the Venn diagrams of all DE analyses illustrate a lack of conservation among DE genes, suggesting a high level of sensitivity and strain-specific expression (**Fig 4**).

While a high number of genes encoding hypothetical proteins were highly DE, so were highly characterized membrane components including LigB, LipL45, and LPS which are known to interact with the host immune response (**Figs 5 and 6**). A subunit vaccine from a conserved portion of LigB has already been established to provide a protective immune response against challenge in the hamster model [42] and additionally, LigA and LigB surface protein expression levels change when *L*. *interrogans* is expanded at 37°C compared to 30°C [43], which suggests a temperature response of interest. Interestingly, in the present study *ligB* was inversely differentially expressed within strain by temperature (upregulated in JB197 at 37°C compared to 29°C and downregulated in HB203 at 37°C compared to 29°C). Collectively this indicates that surface proteins critical to host immune recognition of the pathogen are highly strain specific and can be influenced by environmental temperature. LipL45 is recognized as a membrane lipoprotein which has previously been reported to have been upregulated at 37°C compared to 30°C [37], which is consistent for the data reported here with LipL45 upregulated at 37°C in both JB197 and HB203 compared to 29°C (**Tables 5** and **S4**). This evidence that certain immunogenic surface proteins, such as LipL45, are upregulated at traditional host temperature of 37°C, makes a strong case for testing virulence factors at the newly achievable culture temperature of 37°C with HAN media. Also important is the consideration of culturing bacterins for potential vaccine production at 37°C opposed to the traditional temperature of 29°C, which may increase the expression of certain virulence factors similar to LipL45, which was successful at inducing humoral and cell mediated responses in an early DNA vaccine construct [44]. LPS is recognized as a classic facilitator of pathogen and host immune response interaction. The O-antigen variable portion of LPS, encoded by the *rfb* region, has also been implicated in complement and adhesion mechanisms, and has previously been shown to be differentially expressed by leptospires and state of infection (more greatly expressed by leptospires recovered from the renal tissue of chronically infected rats and weakly expressed in leptospires sourced from acutely ill guinea pig tissue) [45]. Consistent with these results, this study highlights several genes within the *rfb* locus that are significantly differentially expressed when evaluated with a minimum two-fold change (**Table 6**) all being higher in chronic HB203 strain versus the acute JB197 at 29°C. Interestingly, the LPS profile shows the inverse, with higher LPS concentrations detected in JB197 compared to HB203 at both temperatures. It is possible that additional transcriptomic loci have regulatory relationships with the *rfb* region (**S5 Fig**) or alternative genes impact LPS expression. LPS remains an obvious target for vaccine targeted host immunogenicity. Investigation of transcriptomics, proteomics, and LPS not only has implications for the selection of specific strains utilized in companion animal and livestock species vaccine design, but also for selection of temperature at which bacterin sources should be cultured and consistently maintained. Further, investigation of unannotated transcriptomic activity highlights the probability of other regulatory elements, such as sRNAs, or other potential transcriptional regulators, which may play roles in strain specific behavior that will continue to be important to characterize.

Collectively, this study establishes that gene expression is highly specific at the strain level when comparing *Leptospira* even of the same species and serovar. Additionally, gene expression is greatly sensitive to temperature changes similar to those a leptospire may encounter naturally during its life cycle. Characterization of the transcriptome of *L*. *borgpetersenii* serovar Hardjo strains JB197 and HB203 provides insights into factors which may correlate with acute versus chronic disease in the hamster model of infection. The expression of known protective antigens, including LPS and outer membrane proteins such as LigB, can differ significantly between strains, information which needs to be defined when selecting strains for use as bacterin vaccines in domestic animals. Finally, the transcriptome of leptospires cultured at 37°C appears less variable than that of those cultured at 29°C, an additional consideration in bacterin preparation; any additional protective capacity of such bacterin preparations remains to be determined.

## Supporting information

S1 Table Mapping statistics for RNAseq data including the total number of reads sequenced as well as un-mapped, multi-mapped, and uniquely mapped reads. (XLSX)

S2 Table Differential expression of annotated genes for the 29°C contrast (JB197 29°C vs. HB203 29°C). Genes that were considered significantly differentially expressed were required to have an adjusted p-value < 0.05, have a fold change (FC) greater than or equal to three, and a count minimum of at least ten normalized counts in three out of four replicates for a single condition (strain and temperature combination). Significantly DE genes are highlighted in yellow. The first sheet consists of all DE analyses sorted by adjusted p-value, and the second sheet consists of all DE genes sorted by total expression.
(XLSX)

S3 Table Differential expression of annotated genes for the 37°C contrast (JB197 37°C vs. HB203 37°C). Genes that were considered significantly differentially expressed were required to have an adjusted p-value < 0.05, have a fold change (FC) greater than or equal to three, and a count minimum of at least ten normalized counts in three out of four replicates for a single condition (strain and temperature combination). Significantly DE genes are highlighted in yellow. The first sheet consists of all DE analyses sorted by adjusted p-value, and the second sheet consists of all DE genes sorted by total expression.
(XLSX)

S4 Table Differential expression of annotated genes for the JB197 contrast (JB197 29°C vs. JB197 37°C). Genes that were considered significantly differentially expressed were required to have an adjusted p-value < 0.05, have a fold change (FC) greater than or equal to three, and a count minimum of at least ten normalized counts in three out of four replicates for a single condition (strain and temperature combination). Significantly DE genes are highlighted in yellow. The first sheet consists of all DE analyses sorted by adjusted p-value, and the second sheet consists of all DE genes sorted by total expression.
(XLSX)

S5 Table Differential expression of annotated genes for the HB203 contrast (HB203 29°C vs. HB203 37°C). Genes that were considered significantly differentially expressed were required to have an adjusted p-value < 0.05, have a fold change (FC) greater than or equal to three, and a count minimum of at least ten normalized counts in three out of four replicates for a single condition (strain and temperature combination). Significantly DE genes are highlighted in yellow. The first sheet consists of all DE analyses sorted by adjusted p-value, and the second sheet consists of all DE genes sorted by total expression.
(XLSX)

S1 Fig Genome Alignment of JB197 and HB203 for A) Chromosome 1 and B) Chromosome 2. (TIF)

S2 Fig Spearman correlations for four biological replicates of each condition (JB197 29°C, JB197 37°C, HB203 29°C, HB203 37°C) used for RNAseq analysis. (TIF)

S3 Fig Validation of *lipL45*. RNAseq of *lipL45* is visually represented using IGB (**A**). RT-qPCR contrasts are shown for 29°C (JB197 vs. HB203) and JB197 (37°C vs. 29°C) normalized to expression of JB197 29°C and using the *secA* control gene (**B**), or RT-qPCR contrasts are shown, normalized to expression of JB197 37°C utilizing the *rho* control gene for 37°C (JB197 vs. HB203) and HB203 (37°C vs. 29°C) (**C**). * indicates a p-value < 0.05. Error bars represent 95% confidence intervals.
(TIF)

S4 Fig Validation of LBJ_RS02895 and LBJ_RS11060. RNAseq of LBJ_RS02895 as visualized by IGB (**A**) and validated by RT-qPCR normalized to JB197 at 29°C (**B**). RNAseq of LBJ_RS11060 as visualized in IGB (**C**)**,** and validated by RT-qPCR normalized to JB197 at 37°C (**D**). * indicates a p-value < 0.05. Error bars represent 95% confidence intervals.
(TIF)

S5 Fig Non-coding expression in the flanking intergenic regions of select genes within the *rfb* loci visualized with IGB. (TIF)
