# Supplementary figures and images for: Distinct transcriptional profiles of Leptospira borgpetersenii serovar Hardjo strains JB197 and HB203 cultured at different temperatures
Source: PLoS Negl Trop Dis. 2021 Apr 7;15(4):e0009320. doi: 10.1371/journal.pntd.0009320 (PMC8055020; doi:10.1371/journal.pntd.0009320)

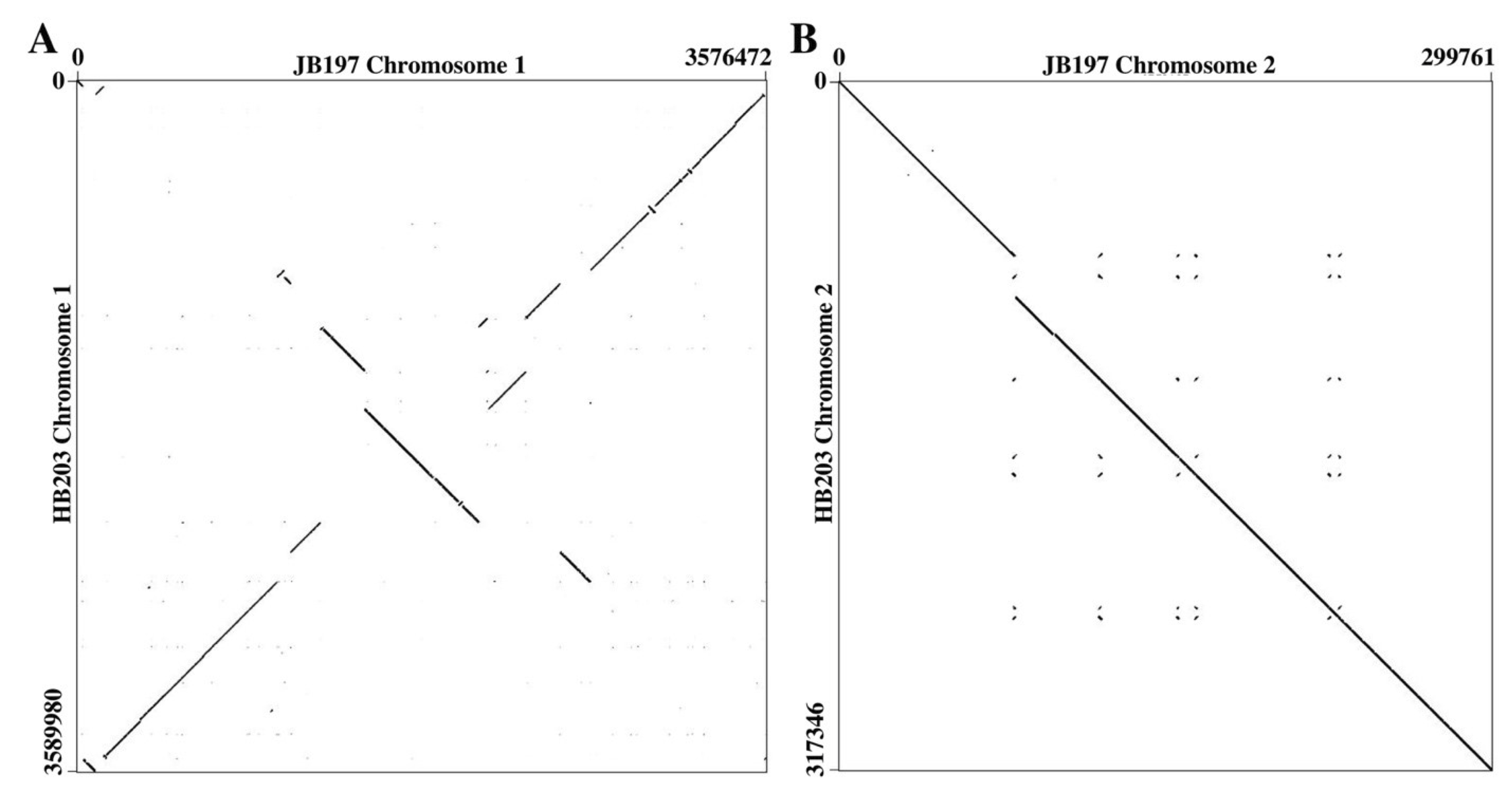

Supplement: S1 Fig — (TIF) [file pntd.0009320.s006.tif]

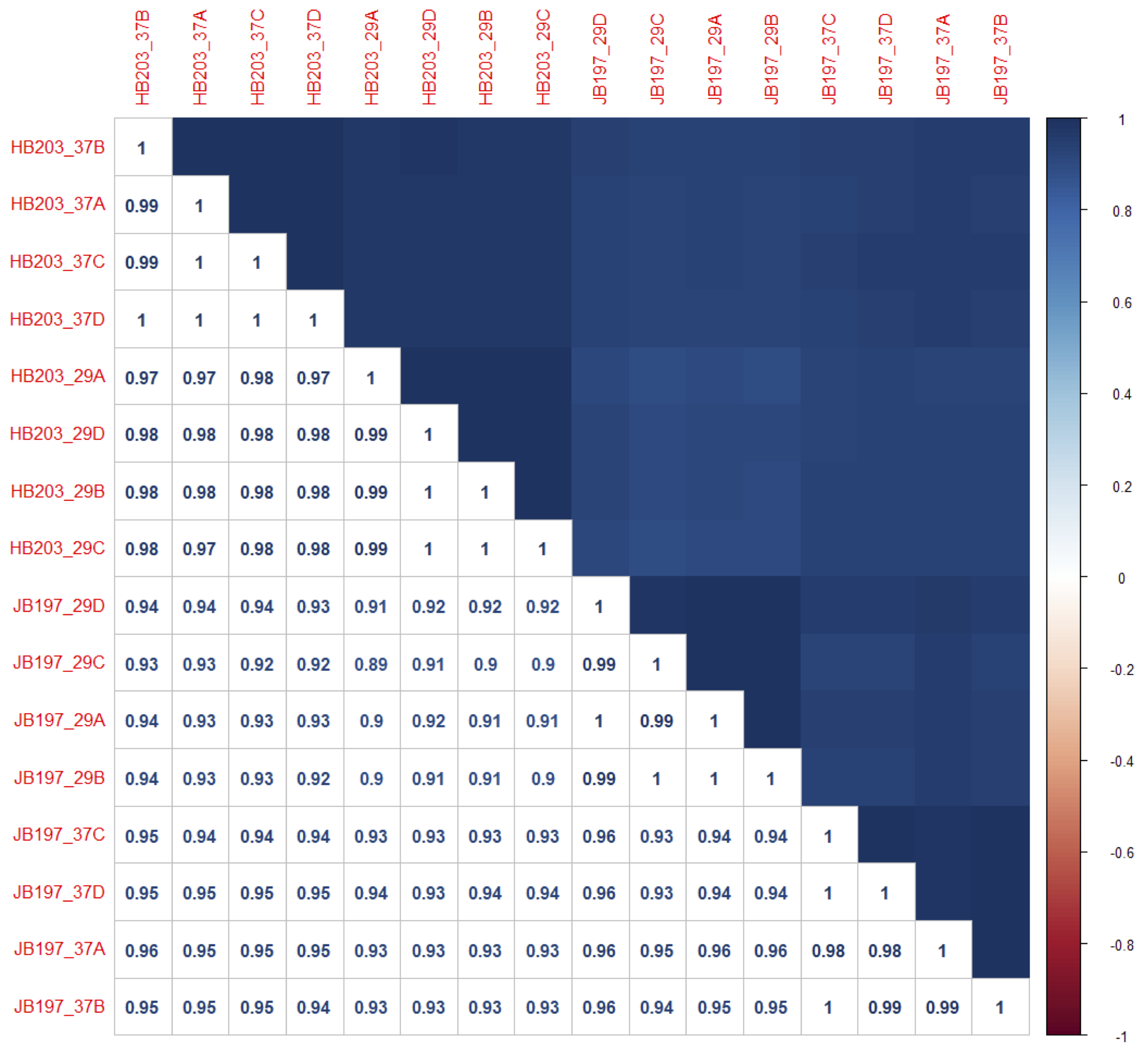

Supplement: S2 Fig — (TIF) [file pntd.0009320.s007.tif]

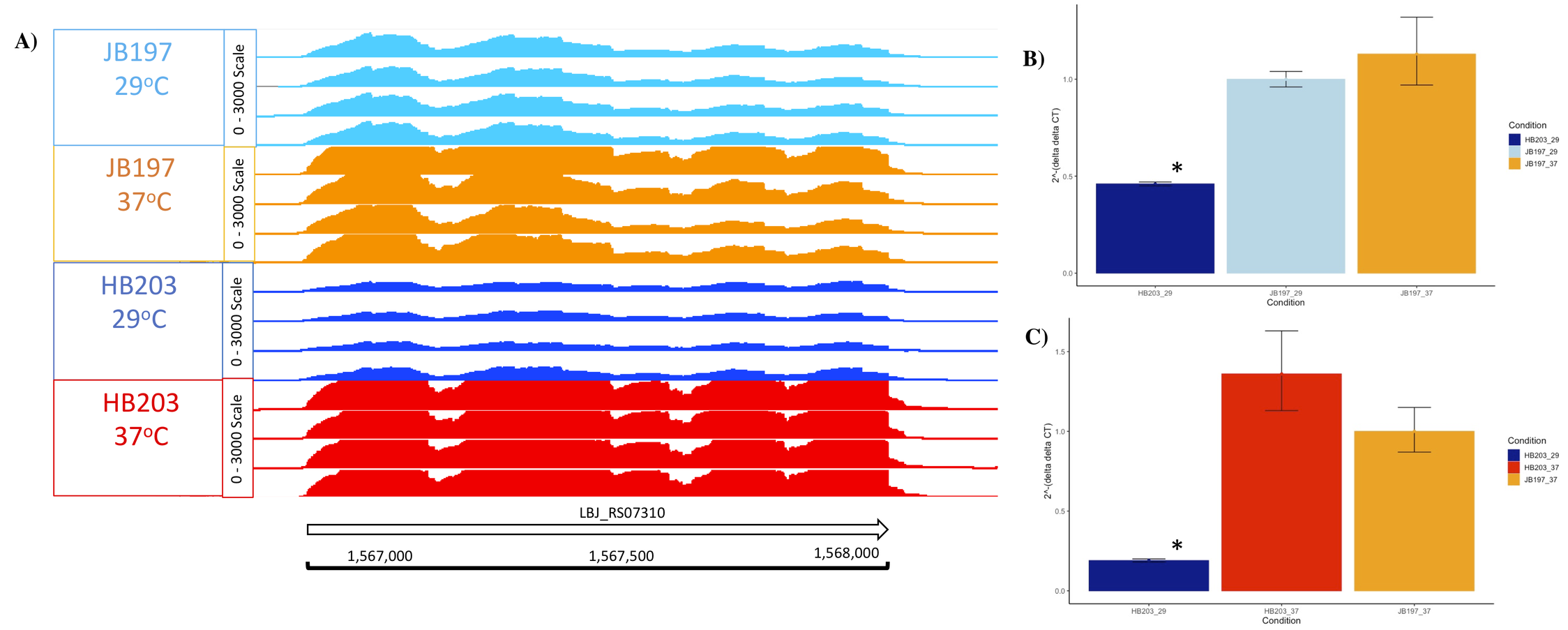

Supplement: S3 Fig — RNAseq of lipL45 is visually represented using IGB (A). RT-qPCR contrasts are shown for 29°C (JB197 vs. HB203) and JB197 (37°C vs. 29°C) normalized to expression of JB197 29°C and using the secA control gene (B), or RT-qPCR contrasts are shown, normalized to expression of JB197 37°C utilizing the rho control gene for 37°C (JB197 vs. HB203) and HB203 (37°C vs. 29°C) (C). * indicates a p-value < 0.05. Error bars represent 95% confidence intervals. (TIF) [file pntd.0009320.s008.tif]

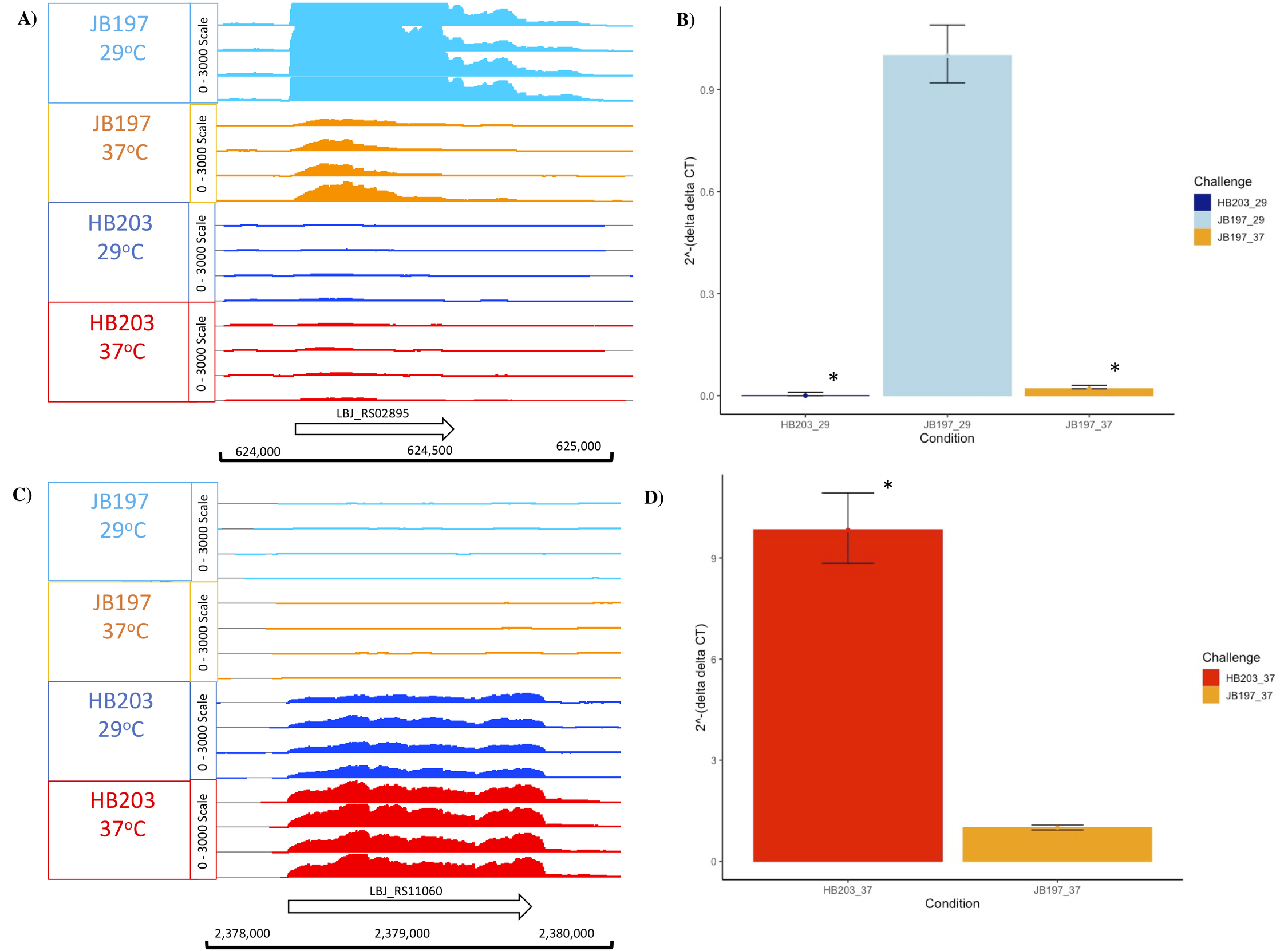

Supplement: S4 Fig — RNAseq of LBJ_RS02895 as visualized by IGB (A) and validated by RT-qPCR normalized to JB197 at 29°C (B). RNAseq of LBJ_RS11060 as visualized in IGB (C), and validated by RT-qPCR normalized to JB197 at 37°C (D). * indicates a p-value < 0.05. Error bars represent 95% confidence intervals. (TIF) [file pntd.0009320.s009.tif]

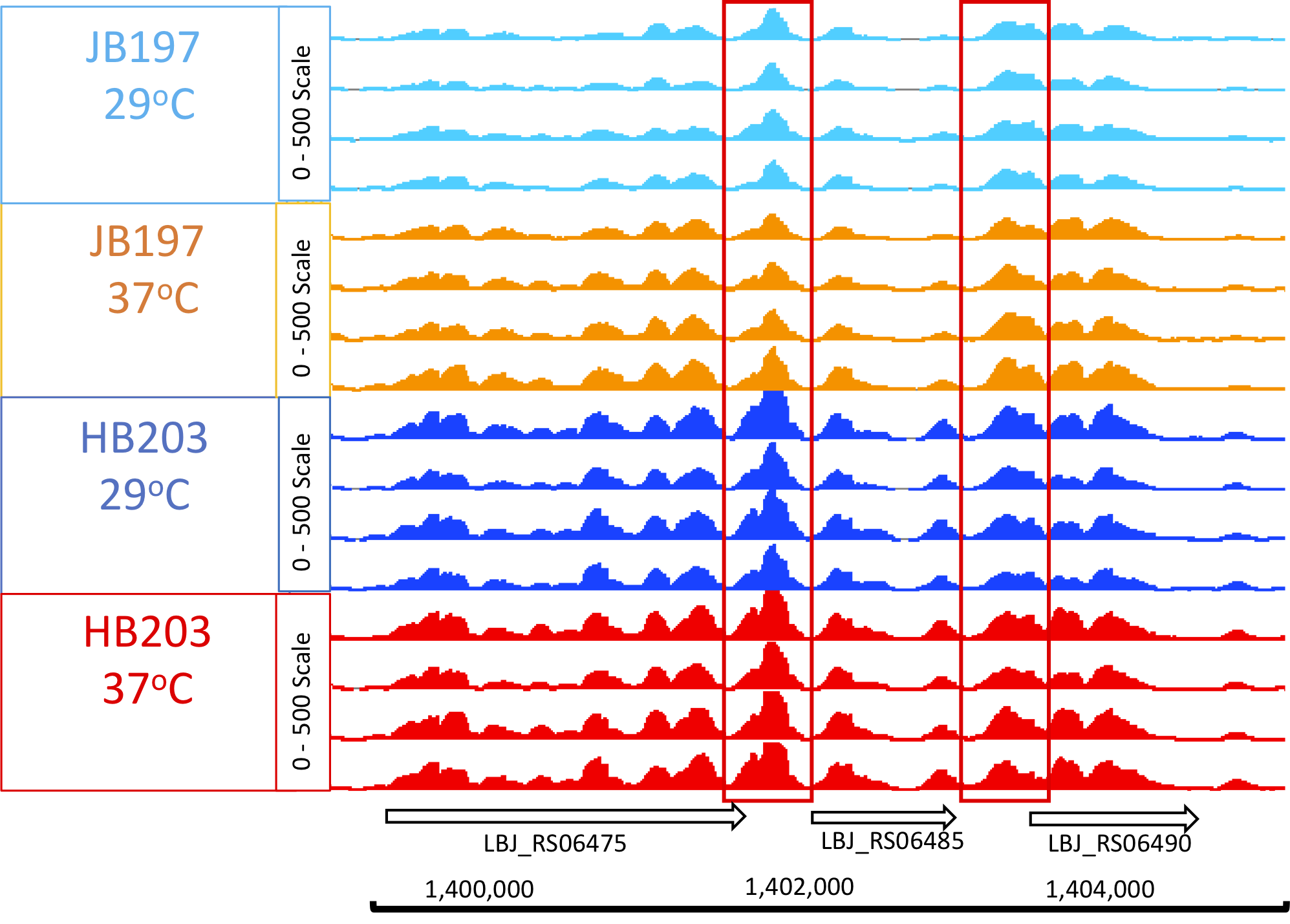

Supplement: S5 Fig — (TIF) [file pntd.0009320.s010.tif]
